# Two-step ATP-driven opening of cohesin head

**DOI:** 10.1038/s41598-017-03118-9

**Published:** 2017-06-12

**Authors:** Íñigo Marcos-Alcalde, Jesús I. Mendieta-Moreno, Beatriz Puisac, María Concepción Gil-Rodríguez, María Hernández-Marcos, Diego Soler-Polo, Feliciano J. Ramos, José Ortega, Juan Pié, Jesús Mendieta, Paulino Gómez-Puertas

**Affiliations:** 1 Centro de Biología Molecular “Severo Ochoa” (CSIC-UAM), 28049 Madrid, Spain; 2 Departamento de Física Teórica de la Materia Condensada and Condensed Matter Physics Center (IFIMAC), Universidad Autónoma de Madrid, 28049 Madrid, Spain; 3 Unidad de Genética Clínica y Genómica Funcional, Departamento de Farmacología-Fisiología y Departamento de Pediatría, Hospital Clínico Universitario “Lozano Blesa”, Facultad de Medicina, Universidad de Zaragoza, ISS-Aragon and CIBERER-GCV02, 50009 Zaragoza, Spain; 4 Departamento de Biotecnología, Universidad Francisco de Vitoria, Pozuelo de Alarcón, 28223 Madrid, Spain

## Abstract

The cohesin ring is a protein complex composed of four core subunits: Smc1A, Smc3, Rad21 and Stag1/2. It is involved in chromosome segregation, DNA repair, chromatin organization and transcription regulation. Opening of the ring occurs at the “head” structure, formed of the ATPase domains of Smc1A and Smc3 and Rad21. We investigate the mechanisms of the cohesin ring opening using techniques of free molecular dynamics (MD), steered MD and quantum mechanics/molecular mechanics MD (QM/MM MD). The study allows the thorough analysis of the opening events at the atomic scale: i) ATP hydrolysis at the Smc1A site, evaluating the role of the carboxy-terminal domain of Rad21 in the process; ii) the activation of the Smc3 site potentially mediated by the movement of specific amino acids; and iii) opening of the head domains after the two ATP hydrolysis events. Our study suggests that the cohesin ring opening is triggered by a sequential activation of the ATP sites in which ATP hydrolysis at the Smc1A site induces ATPase activity at the Smc3 site. Our analysis also provides an explanation for the effect of pathogenic variants related to cohesinopathies and cancer.

## Introduction

Maintenance of the integrity of genomic information is a supreme requirement for all living organisms. In cells, the DNA molecule containing such information is structurally organized in chromosomes, arranged with a number of different protein macromolecular complexes. They are devoted to a variety of functions, from the scaffolding of the chromosomal building to the regulation of the gene expression. The cohesin ring is one of these complexes, an essential nano-machine, powered by ATP hydrolysis, that is capable of encircling DNA strands.

The human cohesin ring is a highly conserved multi-protein structure composed of four major subunits: Smc1A, Smc3, Rad21, and Stag1/2, although only the first three are essential to form molecular rings^1–4^. A heterodimer of Smc1A and Smc3 subunits forms a coiled-coil-structured ring with an ATPase “head” and a “hinge” domain. The C-terminal domain of Rad21 binds to the Smc1A head^5^ while its N-terminal domain binds to the proximal coiled-coil segment of Smc3^6, 7^. The Smc subunits belong to a family of proteins involved in a large variety of functions related to chromosome structure: chromosome segregation during mitosis and meiosis, DNA repair through homologous recombination, organization of the chromatin during interphase and transcription regulation^3, 8–10^.

Defects in the cohesin ring have been related to genetic disorders, known as cohesinopathies, such as Cornelia de Lange Syndrome (CdLS), Roberts Syndrome, Warsaw Breakage Syndrome, CAID Syndrome and CHOPS Syndrome^11–16^, as well as to several types of cancer^17–25^. Defects in proteins that regulate cohesin function have also been related to aneuploidy in neurons, which is a relevant factor in the development of Alzheimer disease^26^.

ATP hydrolysis is required for both DNA loading^27–30^ and unloading^31–33^. An Smc head heterodimer has to be formed, sandwiching the ATP molecules, prior to the hydrolysis event^4, 5, 34^. Although yeast cohesin Smc heads can interact in the absence of Scc1 (the yeast orthologue of human Rad21)^30^, interaction with the Scc1 subunit, in particular with its C-terminal domain, stimulates ATP hydrolysis^27, 35, 36^. The DNA binding-mediated ATPase activity in Smc heads is regulated by the acetylation of several Lys residues located both in the Smc head and in the coiled coils^2, 32, 33, 37, 38^.

As indicated in a recent review^4^, several questions related to the structure and function of cohesin ring remain open. These deal with: the precise series of events that lead to loading, entrapment, release and stable cohesion; the exact role of the nucleotide binding domains; and how ATP binding and hydrolysis affect the loading and release processes. In addition to the biochemical studies and the highly valuable information offered by the crystallized structures of the Smc head domains^5, 7^, it is essential to investigate the dynamic properties at the atomic scale in order to study key aspects of cohesin behaviour as well as to analyse and predict the effect of mutations.

In addition, recent studies^18, 39, 40^ indicate that over-expression of the Smc1A protein can play an important oncogenic role in prostate cancer and colorectal cancer. This suggests that this protein is a promising target for anti-tumour drugs. Detailed study, at the structural and quantitative level, of the transition states as rate-limiting steps in the processes of ATP hydrolysis and the opening of Smc heads is of crucial importance for future rational drug design^41–43^.

**Figure 1:**
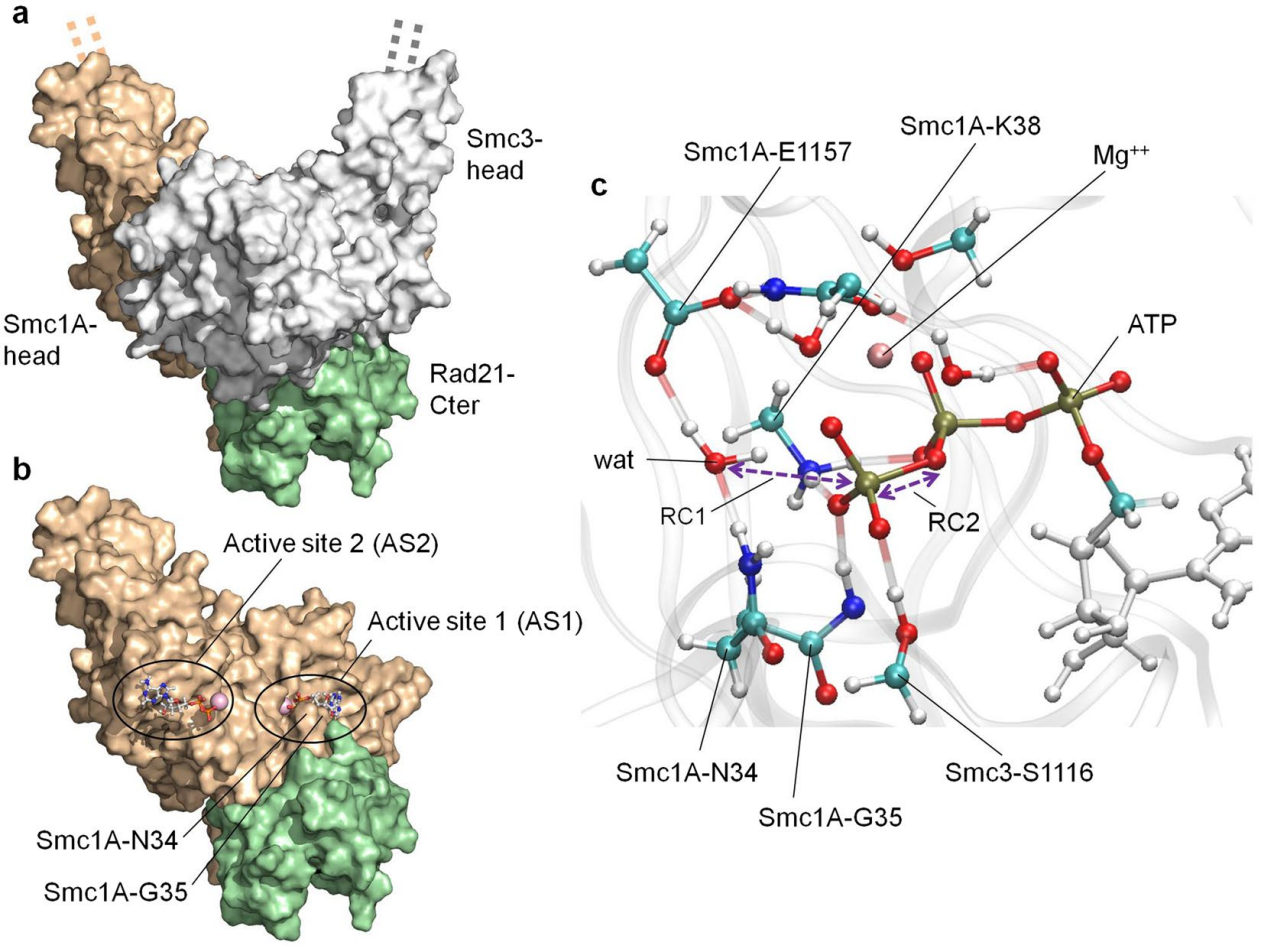
Model overview and description of the QM region. (**a**) Overview of the structural model of the complex formed by the human Smc1A-head (brown), Smc3-head (grey) and Rad21-Cter (green) domains. The dashed lines indicate the direction along which the un-modelled coiled coils would extend towards the hinge domain. (**b**) Location of active site 1 (AS1) and active site 2 (AS2). The Smc1A-head (brown) and Rad21-Cter (green) domains are shown, while the Smc3-head domain is not represented to reveal the location of the active sites. The location of the Smc1A-N34 and Smc1A-G35 residues is indicated. (**c**) QM region of AS1. The atoms in the QM region of the QM/MM MD simulations of AS1 are represented by coloured ball and sticks. The MM regions of the ATP (white ball and sticks) and protein backbone (transparent grey ribbons) are shown. The positions of the catalytic water (wat), residues Smc1A-N34, Smc1A-G35, Smc1A-K38, Smc1A-E1157 and Smc3-S1116, magnesium ion (Mg^++^) and ATP molecule are indicated. Reaction coordinates 1 (RC1) and 2 (RC2) are indicated by purple arrows.

To assess the dynamics of ATP hydrolysis in the cohesin ATPase head heterodimer and its possible effects on the stability of the dimer, we have generated an atomistic model of the ATPase head domains of the human cohesin proteins Smc1A and Smc3 (Smc1A-head and Smc3-head, respectively) bound to the C-terminal domain of human Rad21 (Rad21-Cter) (Fig. 1a). The active site closest to the interface between Smc1A-head and Rad21-Cter, formed by Walker A and Walker B motifs of Smc1A, was labelled as active site 1 (AS1); while the more distant site, formed by Walker A and Walker B motifs of Smc3, was labelled as active site 2 (AS2) (Fig. 1b).

This model mimics the behaviour of the cohesin head, thereby allowing us to investigate and generate functional hypotheses based on detailed analysis of the movement of all the atoms in the head domain; information that cannot be assessed by biochemical assays. In addition, the system enables us to evaluate the role of mutations associated with CdLS and cancer in the functionality of the protein complex.

## Results

### Rad21 binding induces a rearrangement at active site 1 that allows ATP hydrolysis

It has been reported that the dimerized head domains of Smc1A and Smc3 hydrolyse ATP in the absence of Rad21 with a nearly undetectable efficiency, but that when they are bound to Rad21-Cter the hydrolysis activity is enhanced^27, 35, 36^. Using our computational approach, we investigate the hydrolysis reaction at the atomic scale, including the identification of the rate-limiting steps. This analysis provides the explanation for this effect as well as a general model of the movements of all the residues in the complex before and during the ATPase reaction.

In order to evaluate whether the binding of Rad21-Cter directly induces a rearrangement at AS1 that favours ATP hydrolysis, we performed simulations using molecular dynamics and, for the study of the chemical events, our recently developed method for quantum mechanics/molecular mechanics - molecular dynamics (QM/MM MD): Fireball/Amber^44, 45^. This fast and accurate method, combining techniques developed in the areas of computational biology (Amber^46^) and condensed matter physics (Fireball^47, 48^), permits the generation of 2D free-energy maps of enzymatic reactions without *a priori* determination of the reaction paths. In the present case, we simulated ATP hydrolysis at AS1 through the generation of two equivalent systems of the Smc1A-head/Smc3-head dimer, in the presence and absence of Rad21-Cter. Both systems were stabilized with 40 ns of free molecular dynamics (MD) simulations performed with the Amber14 MD package^46^ prior to 150 ps of QM/MM MD stabilization performed using Fireball/Amber. The QM region (Fig. 1c) was formed of the tri-phosphate moiety of the ATP, the magnesium ion, water molecules and side chains present in the coordination sphere of magnesium, the catalytic water molecule and the side chains of Smc1A-N34, Smc1A-G35, Smc1A-K38, Smc1A-E1157 and Smc3-S1116. The MM region comprised the rest of the atoms present in the protein complex and the solvent.

**Figure 2:**
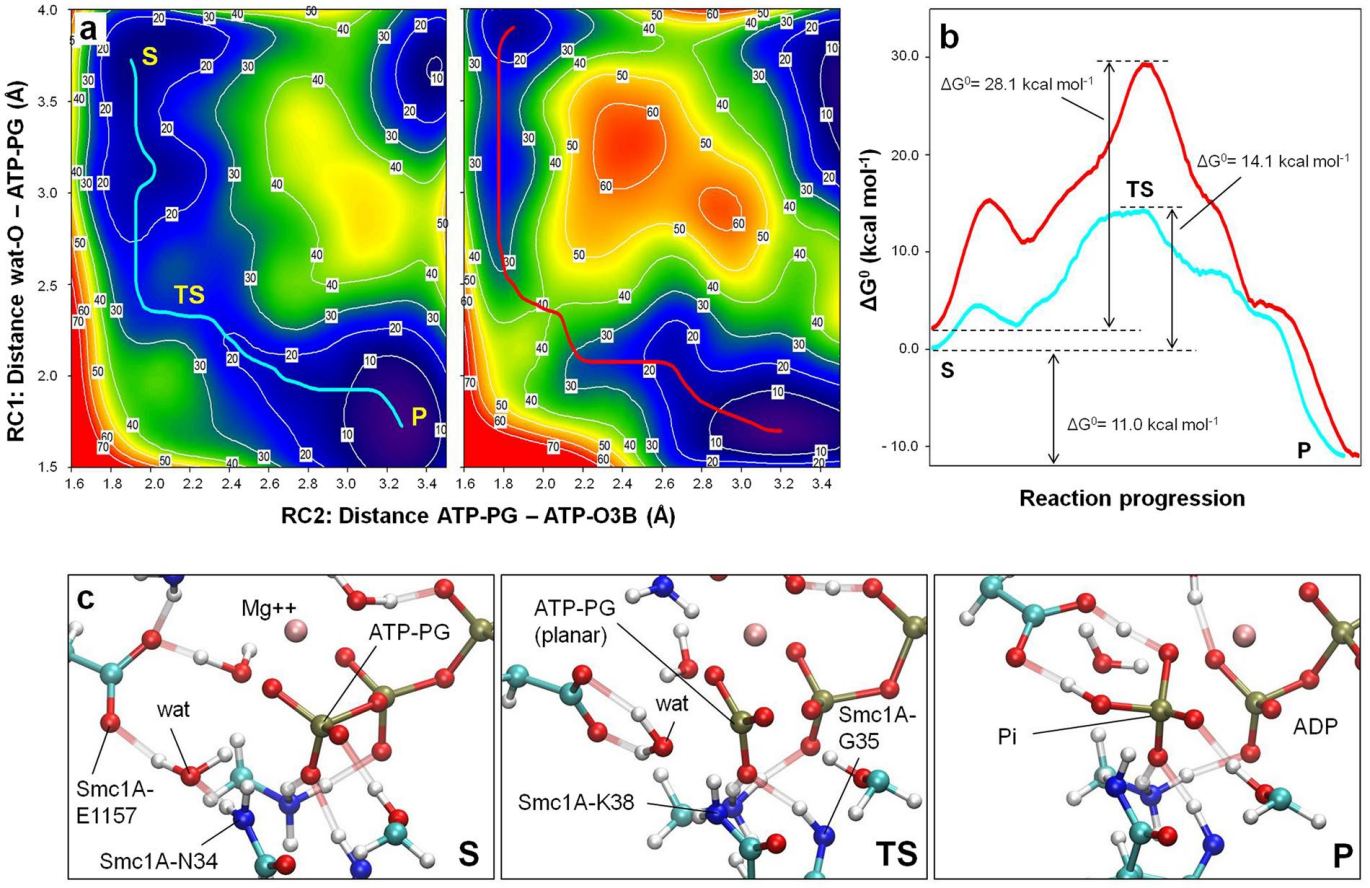
Rad21-Cter allows ATP hydrolysis at AS1. (**a**) Free-energy surfaces (in kcal mol^−1^) for ATP hydrolysis at AS1 in the presence (left) and absence (right) of Rad21-Cter generated via QM/MM MD simulations. The plot axes represent the reaction coordinates. RC1 (bond to be formed): the distance (in Å) between the oxygen atom of the catalytic water and the phosphorous atom of the ATP molecule γ-phosphate group (distance wat-O - ATP-PG). RC2 (bond to be broken): the distance (in Å) between the phosphorous atom of the ATP molecule γ-phosphate group and the oxygen atom 3 of the ATP β-phosphate group (distance ATP-PG - ATP-O3B). Free-energy data are represented via a colour scale, from lower (blue) to higher (red) values. MEPSA minimum energy paths are shown in cyan (presence of Rad21-Cter) and red (absence of Rad21-Cter). (**b**) Free-energy profiles of the MEPSA minimum-energy paths. The substrate (S), transition state (TS) and product (P) locations are indicated. (**c**) The reference structures of S, TS and P states in the presence of Rad21-Cter are shown. The positions of the catalytic water (wat), residues Smc1A-N34 and Smc1A-E1157, magnesium ion (Mg^++^), ATP γ-phosphate (ATP-PG), ADP and leaving inorganic phosphate (Pi) are indicated.

The two free-energy (ΔG°) surfaces were then sampled with Fireball/Amber along two reaction coordinates: reaction coordinate 1 (RC1, the bond to be formed) was the distance between the oxygen atom of the catalytic water and the phosphorous atom of the γ-phosphate group of the ATP molecule while reaction coordinate 2 (RC2, the bond to be broken) was the distance between the γ-phosphate group of the ATP molecule and oxygen atom 3 of the beta phosphate group of ATP (purple arrows in Fig. 1c). The free-energy maps resulting from this sampling (7.6 × 10^6^ conformations and their corresponding total-energies for each map) are depicted in Fig. 2a. In each case, the minimum energy pathway along the calculated free-energy surfaces (cyan and red lines on the maps in Fig. 2a) was calculated using the MEPSA algorithm^49^, by extracting the minimum free-energy path of the reaction from the substrate S (the initial ATP molecule) to the product P (final ADP molecule plus inorganic phosphate group), as represented in Fig. 2b. A detailed explanation of the key reaction steps as well as a video sequence of the whole reaction at AS1 can be found in the Supplementary Information (Supplementary Fig. 1 and Supplementary Video 1).

Briefly, the progression of the ATPase reaction at AS1 in the presence of Rad21-Cter (cyan line in Fig. 2a and b) indicates that the residue Smc1A-N34 plays a crucial role in the entrance of the catalytic water molecule into the active site and its stabilization (Fig. 2c, left), via its coordinated role with the catalytic residue Smc1A-E1157. Smc1A-N34 is maintained in its position by the interaction between Rad21-K605 and Smc1A-G35. The planar structure of the γ-phosphate in the transition state of the reaction (Fig. 2c, centre) is mainly stabilized by Smc1A-K38 and Smc1A-G35 (the latter is also maintained in position by its interaction with Rad21-K605). When the same simulation was run in the absence of Rad21-Cter (red line in Fig. 2a and b), clear differences were observed in the free-energy pathway, which showed higher values during the process of catalytic water accommodation as well as in the transition state (first and second peaks in Fig. 2b). A description of some reaction features at AS1 in presence and absence of Rad21-Cter can be found in the Supplementary Information (Supplementary Figs 2, 3 and 4). The total difference in the free-energy barrier between the two analysed situations was 14.0 kcal mol^−1^ (ΔG° values of 28.1 kcal mol^−1^ and 14.1 kcal mol^−1^ in the absence or presence of Rad21-Cter respectively). In contrast, the ΔG° values of the structures at the beginning (S) and the end (P) of the reaction were almost equivalent in the two situations. This indicates that, regarding ATP hydrolysis at AS1, the main effect of being bound to Rad21-Cter is the reduction of the free-energy barrier. This is in agreement with the experimentally observed fact that the presence of Rad21-Cter allows ATP hydrolysis^27, 35, 36^, lowering the barrier to a calculated ΔG° value close to the range of the experimental free-energy barrier measured for other ATPases, as the F_1_-ATPase (12.9–13.4 kcal mol^−1^)^50^.

All these results indicate that the binding of Rad21-Cter to the Smc1A-head/Smc3-head dimer induces a rearrangement at AS1 that both facilitates the entrance of the catalytic water molecule into the active site and reduces the energy barrier associated with the transition state.

### ATP hydrolysis at active site 1 induces the activation of site 2

Once the ATPase reaction at AS1 was complete, and the site was occupied by the resulting ADP molecule, our next step was to study the effect of this substitution on the structure of the Smc1A-head/Smc3-head/Rad21-Cter complex.

**Figure 3:**
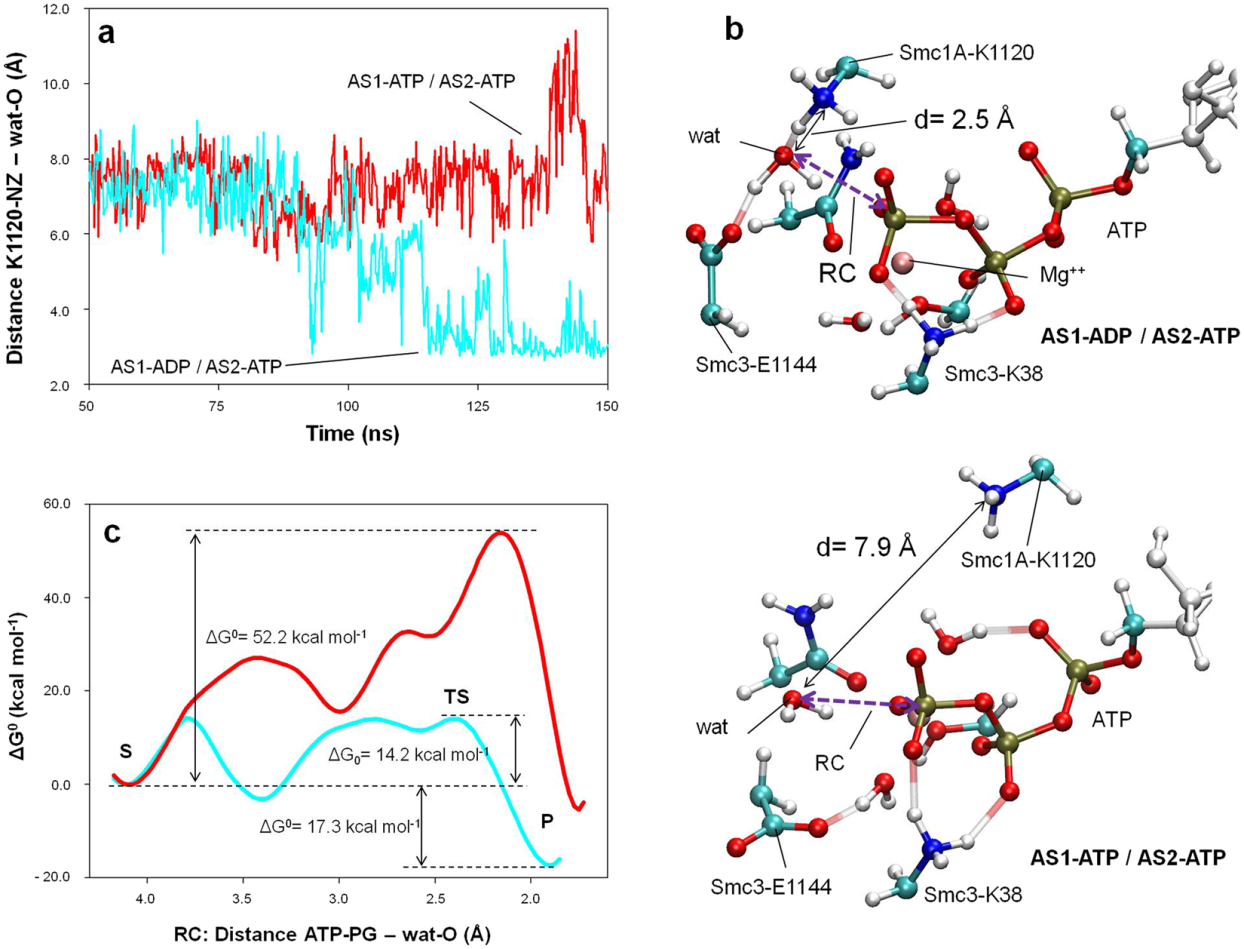
ATP hydrolysis at AS1 activates hydrolysis at AS2. (**a**) Evolution of the distance between the oxygen atom of the catalytic water in AS2 and the ε-amino group of the Smc1A-K1120 residue (distance K1120-NZ - wat-O) prior (red) and after (cyan) ATP hydrolysis at AS1. (**b**) AS2 activation and QM region description. The atoms in the QM region of the QM/MM MD simulations of AS2 are represented by coloured ball and sticks. Part of the MM region of the ATP is shown (white ball and sticks). The positions of the catalytic water (wat), Smc3-K38, Smc3-E1144 and Smc1A-K1120 residues, and the ATP molecule are indicated for both inactive (AS1-ATP/AS2-ATP, red line) and active (AS1-ADP/AS2-ATP, cyan line) AS2 configurations. The distance between the catalytic water and ε-amino group of the Smc1A-K1120 residue is indicated by a black arrow. The reaction coordinate (RC) is indicated by a purple arrow. (**c**) Free-energy (kcal mol^−1^) profiles generated via QM/MM MD simulations of AS2 in both inactive (AS1-ATP/AS2-ATP, red line) and active (AS1-ADP/AS2-ATP, cyan line) configurations. The X-axis represents the reaction coordinate RC (bond to be formed): the distance (in Å) between the oxygen atom of the catalytic water and the phosphorous atom of the ATP molecule γ-phosphate group (distance ATP-PG - wat-O). The substrate (S), transition state (TS) and product (P).

To perform the analysis, the system was subjected to 150 ns of free MD simulation in the presence of an ADP molecule at AS1, while at the same time maintaining an ATP molecule at AS2 (condition AS1-ADP/AS2-ATP). As a control, the same system, but containing ATP at both sites (condition AS1-ATP/AS2-ATP), was subjected to an equivalent simulation. Throughout both trajectories, the movements of residues around the two active centres were monitored for any conformational change that could affect the activity of AS2. Notably, after 120 ns of free MD simulation in the AS1-ADP/AS2-ATP condition, the side chain of an apparently non-related residue, Smc1A-K1120, moved close to the AS2 catalytic water molecule and remained in its new location in a stable conformation (Fig. 3a and Supplementary Video 2). In this condition, the distance between the apical nitrogen atom of the side chain of Smc1A-K1120 and the oxygen atom of the AS2 catalytic water was stabilized at a value of 2.5 Å, in contrast to the value of around 7.9 Å in the AS1-ATP/AS2-ATP condition (Fig. 3b). In the AS1-ADP/AS2-ATP condition, the interaction between Smc1A-K1120 and the catalytic water molecule was found to be stabilized by the formation of a hydrogen bond.

The presence of Smc1A-K1120 in this new position is predicted to dramatically affect the distribution of charges at AS2. To evaluate the extent of this effect, we analysed the ATPase reaction at AS2 in both conditions (AS1-ADP/AS2-ATP and the control AS1-ATP/AS2-ATP) in detail using Fireball/Amber. The initial structures used for both QM/MM MD simulations were the final structures of the 150 ns long free MD simulations shown in Fig. 3a. The QM region (Fig. 3b) was formed of the tri-phosphate moiety of the ATP, the magnesium ion, water molecules and side chains present in the coordination sphere of magnesium, the catalytic water molecule and the side chains of Smc3-K38, Smc3-E1144 and Smc1A-K1120; with the MM region comprising the other atoms in the complex and solvent.

As the entrance of a lysine side chain in close proximity to the catalytic water at AS2 was expected to have a substantial effect on the ATPase activity, the free-energy profiles were initially sampled along a single reaction coordinate, instead of two. The reaction coordinate selected (RC, purple arrow in Fig. 3b) was that corresponding to the bond to be formed: the distance between the oxygen atom of the catalytic water and the phosphorous atom of the γ-phosphate group of the ATP molecule. As expected, 1D free-energy sampling was sensitive enough to detect a remarkable difference between the two conditions. The resulting free-energy profiles for each reaction are depicted in Fig. 3c. A detailed explanation of the key reaction steps as well as a video sequence of the whole reaction at AS2 can be found in the Supplementary Information (Supplementary Figs 3 and 5 and Supplementary Video 3). This QM/MM MD analysis of the reaction revealed the presence of an intermediate transition state during the positioning of the catalytic water molecule, as well as a main transition state corresponding to the planar configuration of the γ-phosphate of the ATP.

Comparison of the 1D free-energy profiles of ATP hydrolysis at AS2 under both conditions showed a reduction of 38.0 kcal mol^−1^ in the energy barrier in the AS1-ADP/AS2-ATP condition compared to the control, with a final value for the reaction barrier of 14.2 kcal mol^−1^ (Fig. 3c). This result reveals that the change in location of Smc1A-K1120, as a result of the presence of ADP at AS1, strongly stimulates the ATPase activity at AS2. In short, our results show that ATP hydrolysis at AS1 induces ATPase activity at AS2 and explain the atomic mechanism for this effect.

### ATP hydrolysis facilitates separation of the ATPase heads

Once both active sites are occupied by ADP molecules, the last step in the analysis must necessarily explore the behaviour of the head dimer in this arrangement. From a biochemical point of view, the need for ATP binding and hydrolysis for both DNA loading^27–30^ and unloading^31–33^ has been described. Also, separation of the ATPase head domain heterodimer, which allows DNA to pass through, is assumed in current models of cohesin function^2, 27, 29–33, 36, 51^. However, the underlying details of this mechanism have yet to be reported in order to allow quantification of the contribution of the ATP molecules to the maintenance of the closed conformation of the ring.

**Figure 4:**
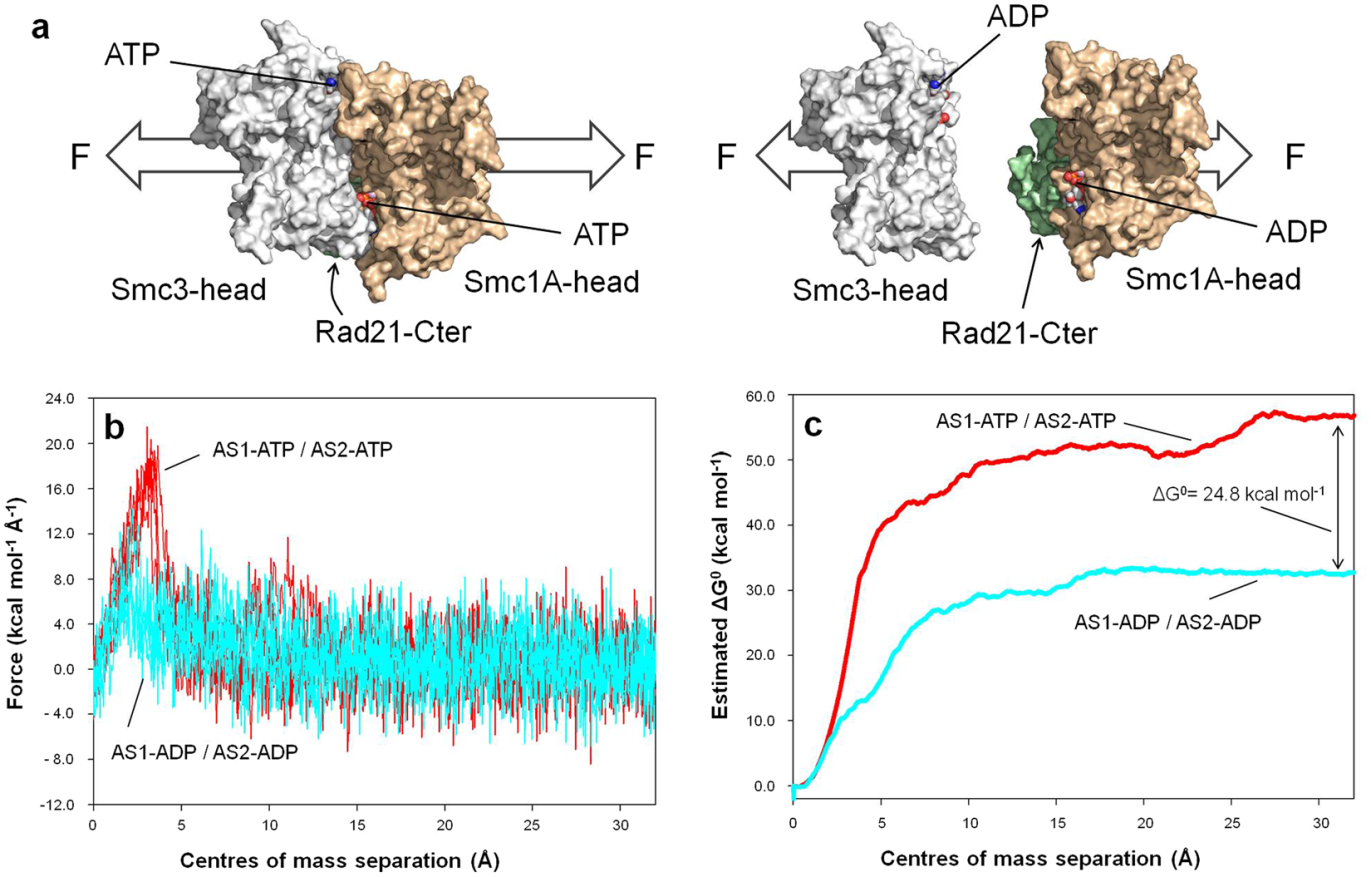
ATP hydrolysis at AS1 and AS2 facilitates head separation. (**a**) Schematic overview of the head separation induced by SMD simulations. The Smc1A-head (brown), Smc3-head (grey) and Rad21-Cter (green) domains are shown and the nucleotide (ATP or ADP) locations in both active sites are indicated. Force (F) direction is marked by white arrows. (**b**) Forces exerted in SMD trajectories over the separation between the centres of mass of Smc1A-head and Smc3-head domains. Points from all trajectories for the AS1-ATP/AS2-ATP condition (red) and for AS1-ADP/AS2-ADP condition (cyan) are shown. (**c**) Estimated free-energy difference (kcal mol^−1^) over the separation between the centres of mass of the Smc1A-head and Smc3-head domains computed using Jarzynski’s equality over 5 SMD trajectories for each condition.

To gain insight into this matter, two equivalent structures of the head complex were generated, one containing ATP at both active sites (AS1-ATP/AS2-ATP condition: the system in a conformation prior to the ATP hydrolysis), and the other containing ADP in both active sites (AS1-ADP/AS2-ADP condition: the system after the hydrolysis events). Both structures were stabilized over 150 ns of free MD simulation. For each condition, 5 individual structures were extracted from the free MD trajectories: one every 4 ns from 104 ns to 120 ns. Using those structures, the separation of the heterodimer head domains was analysed via steered MD (SMD) simulations (Fig. 4a and Supplementary Fig. 6), forcing their centres of mass to separate from each other 32.5 Å over 13.0 ns. The values measured for the accumulated work over the 10 SMD trajectories (5 for each condition) were used to estimate the free-energy difference associated with the opening of the head in both conditions, using Jarzynski’s equality^52^. Jarzynski’s equality allows us to estimate the free-energy difference between two quasi-equilibrium states (in our case, closed and open Smc1A-head/Smc3-head/Rad21-Cter complexes) by collecting the work done over non-equilibrium transitions between those states (in our case, the SMD trajectories).

Force values measured along the head separation in the AS1-ATP/AS2-ATP condition (Fig. 4b, red lines) showed a peak after the first 5 ns (around 3.5 Å of separation between the centres of mass) in all the trajectories. This peak is significantly larger than that observed in the AS1-ADP/AS2-ADP condition (Fig. 4b, cyan lines). In the calculation of the free energy, this force peak represents the largest contribution to the difference between the two conditions: ΔG^0^ = 24.8 kcal mol^−1^. It should be noted that this estimated value is approximately 81% of the average ΔG^0^ associated with the hydrolysis of two molecules of ATP in human resting muscle conditions: ΔG^0^ = 30.6 kcal mol^−1^ ^53^, which suggests that this system is highly efficient from an energetic point of view and supports the idea that ATP hydrolysis at both active sites allows head separation.

### Pathogenic variants and mutants with an associated phenotypic effect

**Figure 5:**
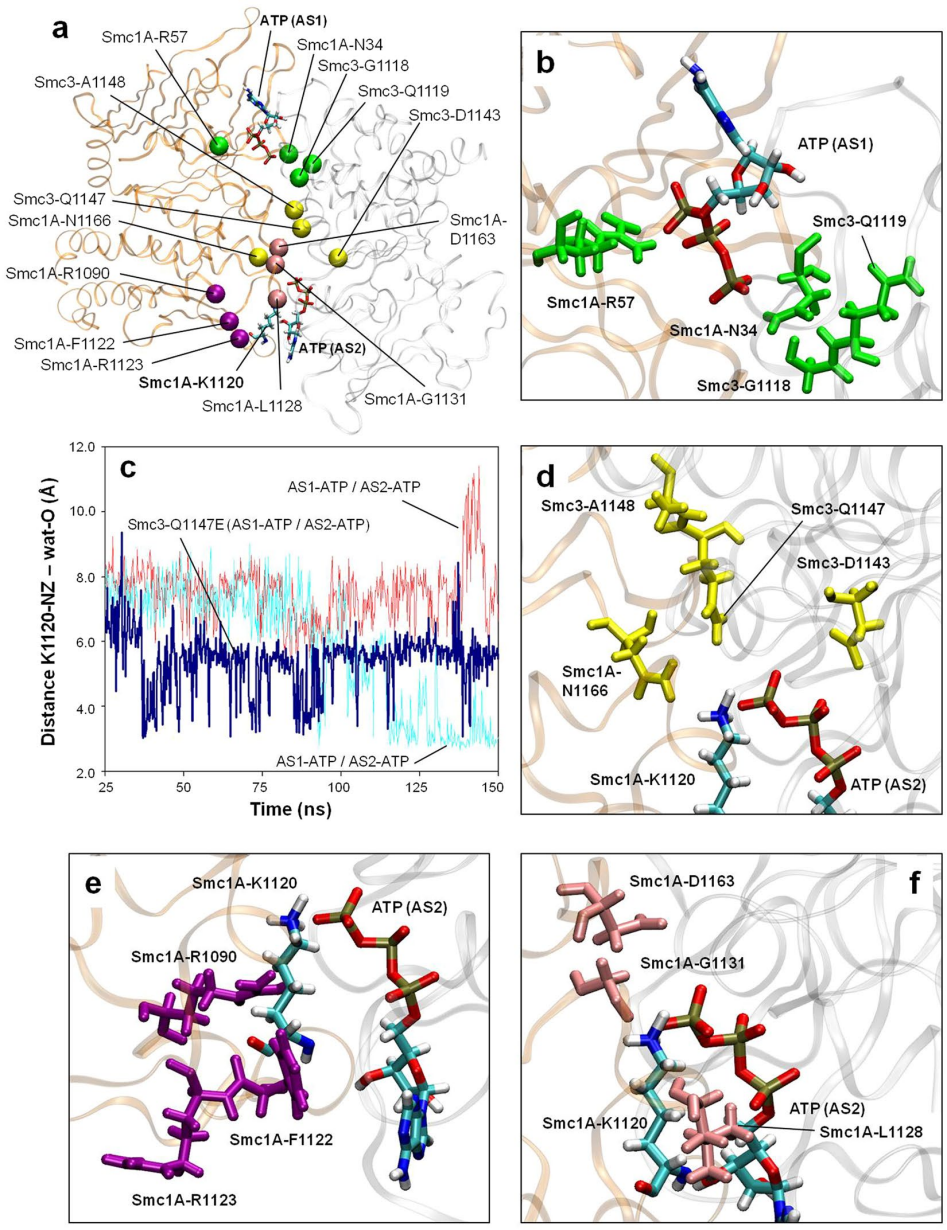
Pathogenic variants. (**a**) Location of the Cα atoms of residues of interest in the neighbourhood of AS1 and AS2. Disease-related variants are shown in green (those affecting AS1), purple (those affecting AS2 activation via Smc1A-K1120 rearrangement) and yellow (those affecting AS2 directly). Residues equivalent to those affected by mutations that bypass the need for Eco1 in yeast are shown in pink. The Smc1A-K1120 residue and both ATP molecules are shown. (**b**) Location of the variants affecting AS1. Residues are depicted in green. (**c**) Evolution of the distance between the oxygen atom of the catalytic water in AS2 and the ε-amino group of the Smc1A-K1120 residue (distance K1120-NZ - wat-O). Distances obtained with wild-type Smc3 prior (red) and after (cyan) ATP hydrolysis at AS1, and distances obtained with the Smc3-N1147E mutant after ATP hydrolysis at AS1 (blue) are shown. (**d**) Location of the variants directly affecting AS2. Residues are depicted in yellow. (**e**) Location of the variants affecting AS2 activation via K1120 rearrangement. Residues are depicted in magenta. The location of K1120 is indicated. (**f**) Location of the residues equivalent to those affected by mutations that bypass the need for Eco1 in yeast. Residues are depicted in pink. The location of K1120 is indicated.

**Figure 6:**
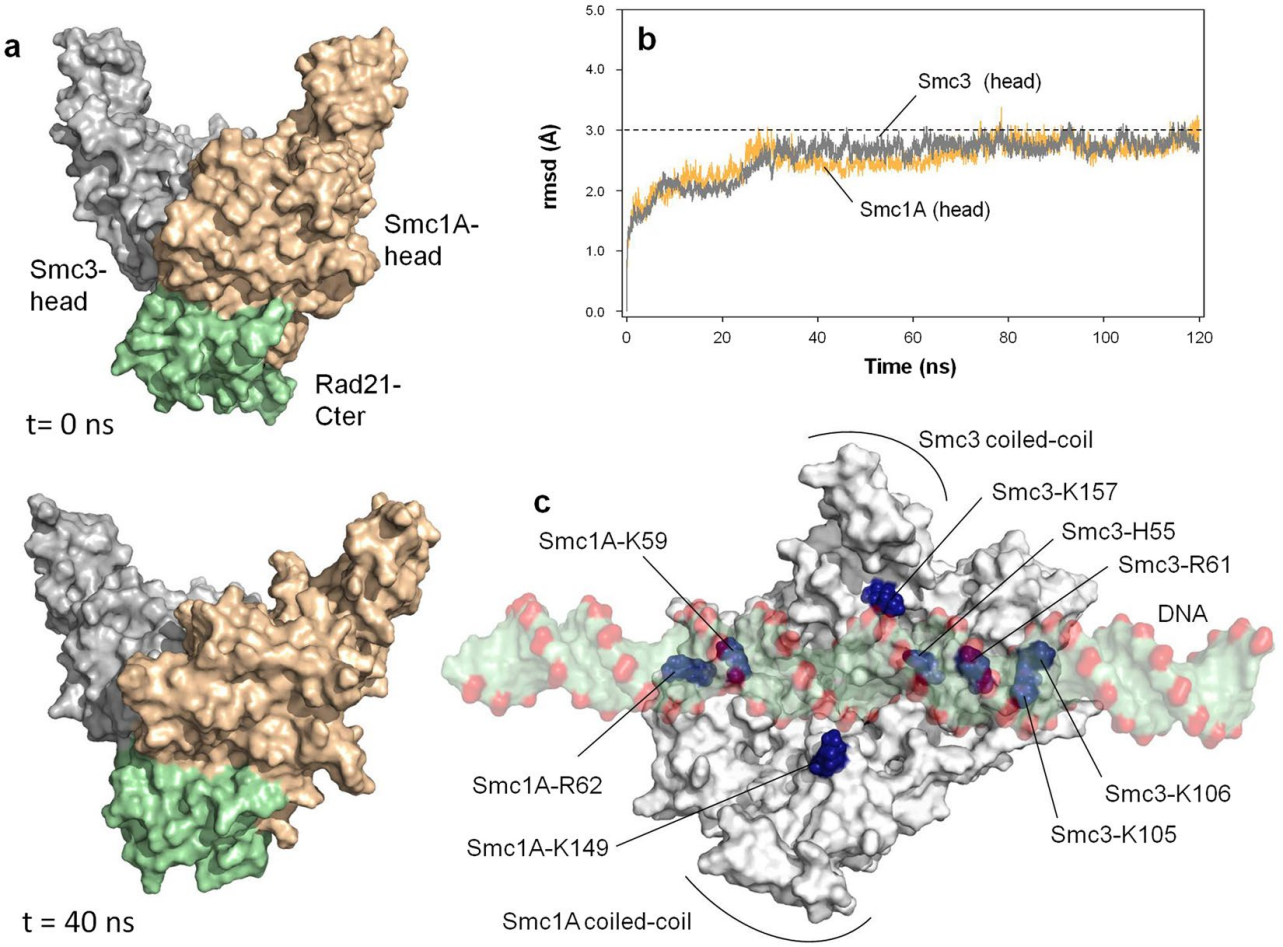
Graphical illustration of a putative interaction of DNA and the head domains of Smc1A and Smc3. (**a**) Relative positions of the Smc1A-head, Smc3-head and Rad21-Cter domains at 0 ns (top) and after 40 ns (bottom) of free MD. (**b**) rmsd values measured over the unrestricted 120 ns MD trajectory of the complex illustrated in **a**. (**c**) Position of positively charged residues in the upper surface of the head complex after 40 ns of MD. The putative position of a DNA molecule, in the equivalent position as the one co-crystallized with Rad50 head domain (PDB code: 5DNY), is indicated.

Our dynamic system at atomic scale of the Smc1A-head and Smc3-head domains provides an advantageous framework for the investigation of pathologies and phenotypic variations associated with specific mutations of residues located in these domains. Table 1 and Figs 5 and 6c summarize this information for some human pathogenic variants as well as for residues whose phenotypic behaviour has been reported in the literature.

**Table 1:** Human pathogenic variants.

Protein	Mutation	Disease	Location	References
Smc1A	N34S	Endometroid carcinoma	Active site 1	54
Smc1A	R57W	Endometroid carcinoma	Active site 1	54
Smc1A	V58_R62del	Cornelia de Lange Syndrome	Putative binding to DNA	9, 55, 58, 59
Smc1A	R1090C	Melanoma	Active site 2 (activation)	20, 54
Smc1A	F1122L	Cornelia de Lange Syndrome	Active site 2 (activation)	9, 59, 74
Smc1A	R1123W	Cornelia de Lange Syndrome	Active site 2 (activation)	59, 63
Smc1A	N1166T	Cornelia de Lange Syndrome	Active site 2	55
Smc3	H55Y	Colorectal cancer	Putative binding to DNA	22, 54
Smc3	G1118V	Acute myeloid leukaemia	Active site 1	21, 54
Smc3	Q1119K	Acute myeloid leukaemia	Active site 1	21, 54
Smc3	D1143H	Acute myeloid leukaemia	Active site 2	21, 54
Smc3	Q1147E	Cornelia de Lange Syndrome (CS: moderate*)	Active site 2 (activation)	12, 56
Smc3	A1148T	Colorectal cancer	Active site 2	54, 57

*The patient Smc3-Q1147E showed craniofacial dysmorphism with brachycephaly, arched eyebrows, a depressed nasal bridge and severe ptosis. Additionally, he had heart abnormalities with atrial and septal defects as well as developmental delays and a learning disability^12^. Clinical severity (CS) has been annotated according to Kline *et al*.^58^.

The residues affecting ATPase activity at AS1 and AS2 can be grouped into four clusters. The first is composed of the residues Smc1A-N34, Smc1A-R57, Smc3-G1118 and Smc3-Q1119 (depicted in green in Fig. 5a and b). The mutations N34S and R57W in human Smc1A have been related to endometrioid carcinoma^54^, with the role of both residues being related to the correct positioning of ATP at AS1. Smc1A-N34 stabilizes the position of the catalytic water during the initial steps of the ATPase reaction (Figs 1c and 2c) and the position of the planar structure of the γ-phosphate during the transition state (Fig. 2c). Smc1A-R57 enters into contact with the α-phosphate group of ATP, thereby stabilizing its position during the entire reaction. Smc3-G1118 and Smc3-Q1119 are located in close contact to Smc1A-R57, allowing for its correct positioning. The mutation of the indicated residues above (depicted in green in Fig. 5a and b) will alter the positioning of ATP at AS1 as well as the progress of the ATPase reaction at this site and subsequent activation of AS2 and head opening.

The second group of residues is composed of the amino acids Smc1A-N1166, Smc3-D1143, Smc3-Q1147 and Smc3-A1148 (depicted in yellow in Fig. 5a and d). The variant residues Smc1A-N1166T and Smc3-Q1147E have been found in patients with CdLS^12, 55, 56^ whereas mutated amino acids Smc3-D1143H and Smc3-A1148T have been related to acute myeloid leukaemia^21, 54^ and colorectal cancer^54, 57^, respectively. The mutant Smc3-Q1147E was previously reported to potentially be involved in maintaining the dimerization contact between the Smc1A-head and Smc3-head domains^12^. Interestingly, in the dynamic model, Smc3-Q1147 was found to be involved in correctly locating residues around Smc1A-K1120. To establish whether the mutated Smc3-Q1147E residue can play a differential role in activation of the AS2 site, the same experiment as the previous one illustrated in Fig. 3a was performed but replacing Smc3-Q1147 by Glu. The result (Fig. 5c) indicated that, during the 150 ns trajectory in the presence of the mutant residue, the distance of Smc1A-K1120 from the catalytic water of AS2 was intermediate between the non-active and the active structures; it was only compatible with an active arrangement for 0.03% of the total time, in contrast to 13.45% in the case of the wild-type Smc3-Q1147 residue. This therefore predicts greatly reduced (but not completely abrogated) ATPase activity at the AS2 site in the cohesin head of the patient. This finding is very exciting as, to the best of our knowledge, this is the first time that a mutated residue from a CdLS patient has been assigned a specific functional role in a dynamic context involving the cohesin complex.

The third group is composed of the Smc1A amino acids: R1090, F1122 and R1123 (depicted in magenta in Fig. 5a and e). The mutant Smc1A-R1090C has been associated with melanoma^20, 54^ and variant residues Smc1A-F1122L^9, 58, 59^ and Smc1A-R1123W^59^ have been found in CdLS patients. The most interesting fact regarding this group of residues is that their positions in the structure are closely related to the movement of Smc1A-K1120 during the activation of AS2. Drastic mutations such as Arg to Trp, in the case of Smc1A-R1123, or to Cys, in the case of Smc1A-R1090C, or more conservative changes such as Phe to Leu in the case of Smc1A-F1122L, can displace Smc1A-K1120 from its correct positioning at the AS2 site, leading to protein malfunction.

The fourth group of residues is composed of the orthologous positions in human sequences of residues that are mutated in yeast and can bypass the need for Eco1: Smc1A residues L1128, G1131 and D1163 (coloured pink in Fig. 5; equivalent to yeast Smc1 residues L1129, G1132 and D1164, respectively^31^). The position of Smc1A-G1131 and Smc1A-D1163, close to AS2, is compatible with differences in functionality when the Gly residue is mutated to Ser or when the Asp residue is mutated to Glu or Gly^31^. More interesting, however, is the case of Smc1A-L1128, as the mutation of the orthologous Smc1-L1129V in yeast affects the off-rate of cohesins but the equivalent mutation in yeast Smc3-L1126V does not. In our simulations, the Smc1A-L1128 residue stabilizes the hydrocarbon side chain of Smc1A-K1120, thereby allowing its correct location and thus leading the ATPase reaction at AS2. Due to the shorter side chain of Val compared to Leu, such hydrophobic stabilization cannot be maintained in the case of the mutant. In contrast, the equivalent residue Smc3-L1115, although located near AS1, does not play such a central role in the reaction, which explains why the conservative mutation of Leu to Val does not result in a differential phenotype. This asymmetric role of Smc1A-L1128 and Smc3-L1115 in our simulations is in agreement with the differential role of the two equivalent residues in yeast^31^.

An additional group of residues of exceptional importance for cohesin function are those related to acetylation-regulated DNA binding. These residues are located both in coiled-coils^37^ and in the inner side of the head domains of the Smc1A-Smc3 dimer^2, 32, 33, 38^. During the initial free MD equilibration of the head structure in our model, and possibly due to the lack of constriction forces exerted by the absent coiled coils in the simulation, the relative angle between the head domains grew wider after 40 ns of MD (Fig. 6a), resembling the open structure of the Rad50 head domain associated with DNA^60–62^. To ensure that such movement did not affect the internal structure of either domain, root mean square deviation (rmsd) values were measured during the unrestricted 120 ns MD trajectory of the complex (Fig. 6b). Despite the indicated movement of the head domains, the rmsd values of each domain remained constant (below 3.0 Å). During the relaxation, a number of positively charged residues spontaneously became located in the surface of the dimer (Fig. 6c): Smc1A residues K59, R62 and K149, and Smc3 residues H55, R61, K105, K106 and K157. In addition to the presence of Smc3-K105 and Smc3-K106, already involved in acetylation-regulated DNA binding^2, 32, 33, 38^, the presence of three additional amino acids in this group is noteworthy: Smc1A-K59 and Smc1A-R62, deletion of which has been related to CdLS^55, 59, 63^ and Smc3-H55, mutation of which to Tyr has been associated with colorectal cancer^22, 54^.

Notably, all these positive residues are positioned in a spatial arrangement that is fully compatible with the putative location of a negatively-charged DNA molecule in the surface (Fig. 6c), equivalent to that observed in the case of Rad50^60–62^. This suggests that the initial structure of the dynamic model may mimic the initial position in which not only is the C-ter domain of Rad21 bound to the head domain of Smc1A, but also the positive residues in the head surface of Smc1A and Smc3 are in a position equivalent to the DNA-bound structure; that is, the starting event in the ATPase-dependent opening of cohesin head.

## Discussion

**Figure 7:**
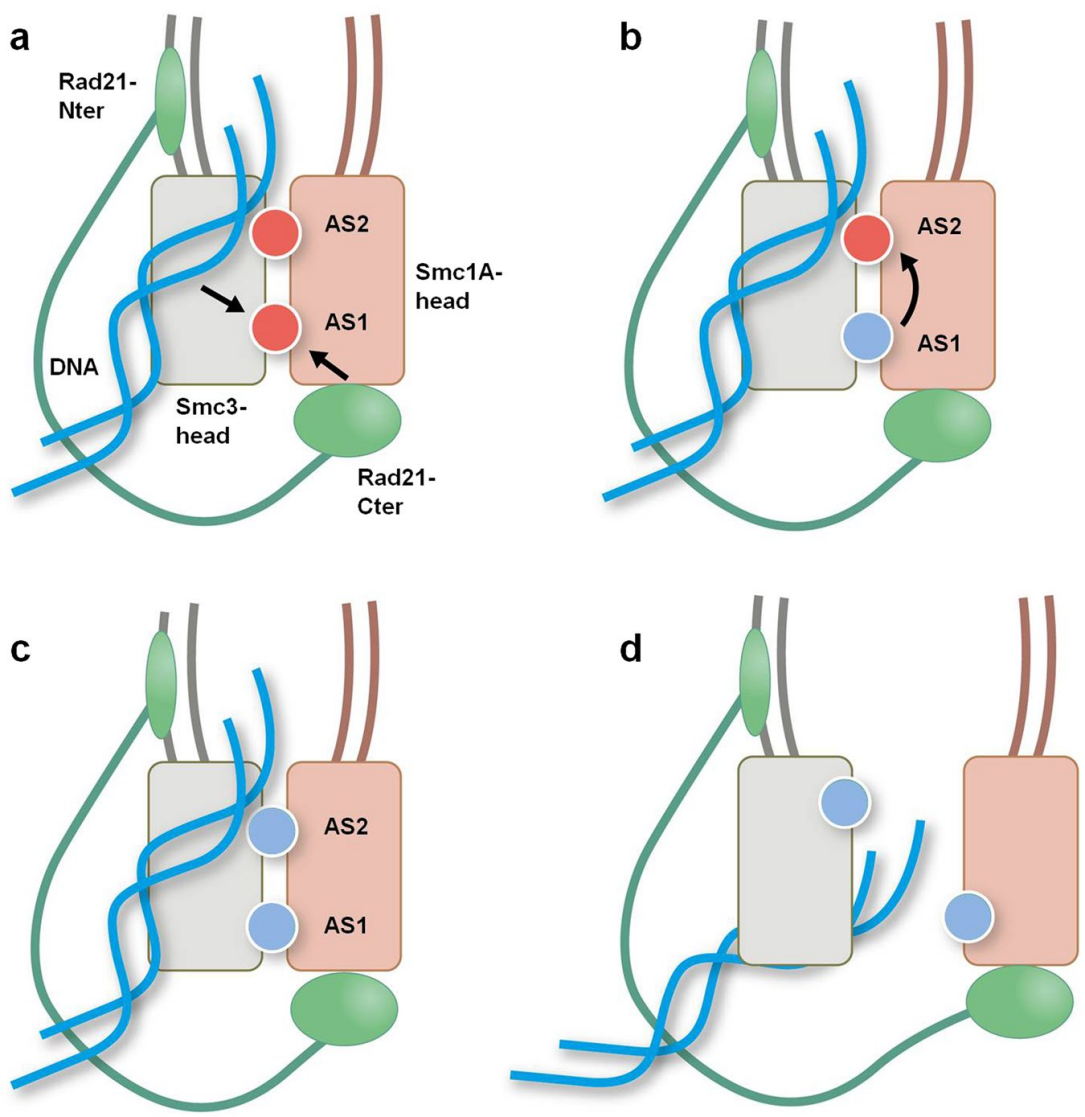
Schematic model for ATP hydrolysis-driven head opening. (**a**) The Rad21-Cter domain binding to the Smc1A-head domain allows hydrolysis at AS1. (**b**) ATP hydrolysis at AS1 induces AS2 activation via Smc1A-K1120 rearrangement. (**c**) ATP hydrolysis takes place at AS2. (**d**) ATP hydrolysis at both active sites facilitates the separation of the ATPase head domains.

Despite the demonstrated relevance of cohesin and cohesin-related proteins to the modulation of important cell functions, the detailed molecular mechanisms underlying the behaviour of the different domains of cohesin proteins has only just begun to be described. In this work, we have analysed the dynamic properties of the human cohesin head domains (Smc1A-head, Smc3-head and Rad21-Cter) at the atomic level using a variety of simulation techniques (free MD, SMD and QM/MM MD). This analysis allows us to infer the role of these domains during ATP hydrolysis events and at the same time to determine how these events affect the atom distribution and function of the protein domains, in a series of events leading to head separation and the subsequent passing of DNA through the open structure (as summarized in Fig. 7).

The first step in the simulation was to describe the role of Rad21 in the hydrolysis of ATP (Fig. 7a). The X-ray structure of the yeast Smc1 head domain bound to the C-terminal domain of Scc1 (the yeast homolog of human Rad21)^5^ offered very detailed information relating to this contact, as the surface between the two domains is located in the neighbourhood of AS1. In our system, the simulated protein complex underwent spontaneous rearrangement resulting in the exposure of a group of positive residues in the inner surface of the Smc1A-head/Smc3-head dimer (Fig. 6c). This group includes two Lys residues proposed as involved in acetylation-regulated DNA binding^2, 32, 33, 38^, as well as other positive residues mutation of which has been related to CdLS^55, 59, 63^ and cancer^22, 54^. In short, the starting point of our simulation corresponds to the head structure bound to Rad21-Cter, in a position compatible with the DNA-bound condition. Our QM/MM MD free-energy analysis for ATP hydrolysis at AS1 provides a quantitative description of the functional role of the Rad21-Cter domain in the cohesin head. The binding of Rad21-Cter allows progression of the ATPase reaction at AS1 by reducing the free-energy barrier by 14.0 kcal mol^−1^. In our study of this reaction we have also determined the geometry of the residues in the active site during critical events, such as the location of the catalytic water in a first intermediate transition state or stabilization of the ATP gamma phosphate group in the planar transition state (see Supplementary Fig. 1 and Supplementary Video 1 for more details).

Once the ATP hydrolysis reaction has taken place at AS1, an intriguing question is whether hydrolysis at one active site can stimulate the ATPase reaction at the other, a common mechanism of ATP-binding cassette-ATPases (ABC ATPases^64^). Our analysis reveals a sequential structural change, after the hydrolysis event at AS1, that connects the two sites (Fig. 7b), in agreement with the fact that the two ATPase sites are asymmetric^31, 51, 65^. Although the primary “driving force” leading to the changes in AS2 is still elusive, it is notorious the fact that the variants implicated in CdLS and cancer are roughly located in the pathway that connects AS1 to AS2 (Fig. 5a). We find Smc1A-K1120 to be a major actor in this process. Smc1A-K1120 is conserved in all Smc1A and Smc1B sequences, as well as in the majority of SMC proteins. Interestingly, an exception to this observation is the condensin subunit Smc2, where the Lys residue in this position is replaced by Thr or Ile in different organisms (Supplementary Fig. 7). In condensin, the Smc2 protein plays the equivalent role to Smc3 in cohesin^1, 8^. Supposing that the behaviour of AS1 and AS2 is similar in cohesin and condensin dimers, then a Lys residue in this position of Smc2 is not necessary for the ATPase activity. Asymmetrically, the Lys residue in the condensin Smc4 subunit (human Smc4-K1183, equivalent to Smc1A-K1120 in cohesin) is indeed conserved. Our QM/MM MD free-energy analysis of the ATPase reaction at AS2 shows that the new structure of the active centre after the movement of Smc1A-K1120 to a position close to the catalytic water results in a very important reduction of the free-energy barrier. As in the case of AS1, our QM/MM MD investigation also provides a detailed description of the hydrolisis reaction at AS2, including all the intermediate steps (see Supplementary Fig. 5 and Supplementary Video 2), from the initial location of the catalytic water molecule to the stabilization of the transition state and the formation of the final product.

The analysis of protein features linked to the structure of the transition state is a key step in the design of transition state analogues as powerful enzymatic inhibitors^41–43^. In this study, using QM/MM MD techniques, we have obtained a detailed description of AS1 and AS2 during the main transition state as well as during intermediate transition states of the ATPase reactions. The geometry of the active centres of the cohesin head in these transient states is extremely valuable information for future drug development strategies.

After the ATP hydrolysis events at AS1 and AS2, we analysed the subsequent head separation (Fig. 7c), including its quantification in terms of free energy. The free-energy difference for the separation of the Smc1A-head and Smc3-head domains, calculated for the ATP/ATP or ADP/ADP conditions, confirms the key role of the two ATP molecules for the stability of the complex. The data suggest that this process is highly efficient from an energetic point of view and support the hypothesis that the hydrolysis of ATP is followed by the opening of the head. This is in agreement with recent observations of a modelled dimer of the yeast Smc1/Smc3 head^36^ as well as recent structural studies of the homologous bacterial SMC dimer^66^.

Finally, the dynamic model generated by mixing different (quantum and classical) simulation strategies at atomic scale is a useful framework within which to rationalize the effect of specific mutations involved in both CdLS and cancer; some for the very first time. In particular, the effect of the variant Smc3-Q1147E, found in a CdLS patient^12, 55, 56^ has been analysed in detail, offering a functional explanation for the impaired behaviour of the protein and linking the change at this position to a defect in the activation of AS2 after activation of AS1. The highly reduced, but not completely inhibited, functionality of the complex could justify the clinical findings in this patient who was phenotypically classified as *moderate* according to Kline *et al*.^58^.

Our study suggests a functional role in DNA binding of three positively charged residues (Smc1A-K59, Smc1A-R62 and Smc3-H55) mutations of which have been related to CdLS^9, 55, 58, 59^ or colorectal cancer^22, 54^. In addition, based on our computational approach, we propose a dynamic explanation for several mutants found in yeast that bypass the need for Eco1^31^. Very interestingly, the close interaction of human Smc1A-L1128 with the key residue Smc1A-K1120 and the lack of a symmetrical interaction in the case of the equivalent residue Smc3-L1115 offer a plausible explanation for the asymmetrical effect found^31^ between the phenotypes of the orthologous yeast mutants Smc1-L1129V and Smc3-L1126V, respectively.

Altogether, our results reveal the underlying atomic mechanisms of the human Smc1A-head/Smc3-head/Rad21-Cter complex, explaining in detail: (i) the functional role of Rad21-Cter in the activation of AS1; (ii) the modifications that link ATP hydrolysis at AS1 with activation of ATP hydrolysis at AS2; and (iii) the role of ATP hydrolysis in the separation of the heads, in a quantitative manner. This computational approach, mixing quantum and classical simulation techniques, has proved to be a very useful tool for the investigation of phenotypic variants in yeast experiments, to analyse and predict the effect of variants and mutants related to CdLS and cancer, and for use in the future design of therapies and drugs.

## Methods

### Structure modelling

The three-dimensional model of the complex formed by the human Smc1A-head, Smc3-head and Rad21-Cter domains was built through modelling procedures using the initial protein sequences contained in the UniProtKB database. They are the Smc1A-head: SMC1A_HUMAN (UniProt code: Q14683, residues 1 to 175 and 1058 to 1223); Smc3-head: SMC3_HUMAN (UniProt code: Q9UQE7, residues 1 to 179 and 1045 to 1206); and Rad21-Cter: RAD21_HUMAN (UniProt code: O60216, residues 543 to 629). Three scaffold structures were combined to accurately reproduce various structural features. Rad21-Cter, the active sites (AS1 and AS2) and the interaction interfaces were modelled on the structure of a *Saccharomyces cerevisiae* homodimeric Smc1 ATPase head complex bound to the C-terminal domain of the yeast Scc1(Rad21 orthologue) (Protein Data Bank ID: 1W1W^5^). The structure of Smc3-head was determined by the 3D structure of the *Saccharomyces cerevisiae* Smc3 monomer bound to the Scc1 N-terminal domain (PDB ID: 4UX3^7^). The model of the complex is compatible with recent structures of human cohesin head, obtained by using high-resolution electron microscopy^67^, and bacterial SMC head, obtained by crystallography^66^. Multiple sequence alignment of the modelled sequences can be found in the Supplementary Information (Supplementary Fig. 8). The positions of residues around the active sites as well as those of crystallographic water molecules were also refined using the 3D structure of the *Pyrococcus furiosus* Smc homodimer (PDB ID: 1XEX^34^). The ATPγS molecules present at 1 W1W active sites were replaced by either ATP or ADP. The model of human variant Smc3-Q1147E was obtained by replacing the apical amide group in the Smc3-Q1147 residue by a carboxylate group.

### Free Molecular Dynamics simulations

Prior to any other simulations, the complex was thermalized and stabilized with free MD simulations using the AMBER14 molecular dynamics package^46^. The 3D structures were solvated with periodic cuboid pre-equilibrated solvent boxes of TIP3P model water molecules^68^ using the LEaP module of AMBER, with 12 Å as the shortest distance between any atom in the protein and the periodic box boundaries. Protonation states were determined using the H++ web server (http://biophysics.cs.vt.edu/H++)^69^ and Na^+^ counterions were added to neutralize the charge of the systems (Smc1A-head/Smc3-head/AS1-ATP/AS2-ATP: 5 Na^+^ counterions; Smc1A-head/Smc3-head/Rad21-Cter/AS1-ATP/AS2-ATP: 3 Na^+^ counterions; Smc1A-head/Smc3-head/Rad21-Cter/AS1-ADP/AS2-ATP: 2 Na^+^ counterions; Smc1A-head/Smc3-head/Rad21-Cter/AS1-ADP/AS2-ADP: 1 Na^+^ counterion). All the free MD simulations were performed in the NPT (constant temperature, constant pressure) ensemble, using the PMEMD program of AMBER and the parm99 force field^46^. The SHAKE algorithm was used, allowing a time step of 2 fs.

The different systems used in the simulations were initially relaxed over 15,000 steps of energy minimization with a cut-off of 12 Å. Then the MD simulations were started with a 20 ps heating phase in which the temperature was raised from 0 to 300 K in 10 temperature change steps, after each of which velocities were reassigned. During minimization and heating, the Cα trace dihedrals were restrained with a force constant of 500 kcal mol^−1^ rad^−2^ and gradually released in an equilibration phase in which the force constant was gradually reduced to 0 over 200 ps. After the equilibration phase, 120 to 150 ns of productive MD simulations were obtained for all the systems.

As the Smc1A-head and Smc3-head domains are formed by the N-terminal and C-terminal segments of the two proteins, all the structures showed gaps where the large coiled-coil regions cannot be accurately modelled. Therefore, these gaps were protected by distance restraints to prevent artificial unfolding of these regions.

During long MD trajectories, and in order to improve the sampling of catalytic configurations at both active sites, the position of the catalytic water molecules was maintained in a geometry compatible with hydrolysis, restraining the distance between the oxygen atom of the catalytic water and the phosphorous atom of the ATP γ-phosphate group below 3.5 Å. The angle between these same two atoms and the oxygen atom of the ATP beta phosphate group was kept between 160° and 180°. Both distance and angle restraints were defined using a flat-bottomed potential, allowing free movement within the restrained range. These restraints were released prior to QM/MM MD simulations of the active centres.

### QM/MM MD simulations

QM/MM MD simulations were performed using the recently developed Fireball/Amber method^44, 45^: a combination of the AMBER molecular dynamics package^46^ and Fireball, a local-orbital density-functional theory molecular dynamics technique^47, 48^. Two regions (QM and MM) were defined. The MM region was treated in the same manner as in the free MD simulations detailed previously; while the QM region was described using Fireball, with a basis set of optimized numerical atomic-like orbitals (NAOs) with a single *s* orbital for H, *sp3* orbitals for C, N and O, and *sp3d5* orbitals for P, as used in previous works^44, 70^. The time step during these calculations was 0.5 fs. The initial structures and initial velocities used in the QM/MM MD simulations were taken from the free MD simulations after they became stable.

Free-energy 2D maps obtained using QM/MM MD simulations were generated as described in the literature^44, 70, 71^. The conformational space was sampled with long SMD trajectories along the chosen reaction coordinates, generating 7.6 × 10^6^ structures with their associated reaction coordinates and energy values. The QM energy values were distributed in groups of ~1.5 × 10^4^ different structures on average, across a uniform grid defined by the two reaction coordinates. The partition function was calculated for each group in order to estimate a free-energy surface that was then smoothed via a 3D LOESS local regression. Reaction paths and energy profiles were calculated using MEPSA^49^.

1D free-energy profiles were generated by sampling initial structures along the reaction coordinate via SMD. These structures were then relaxed for 5 ps by keeping the reaction coordinate fixed at the corresponding value. Velocities were reassigned every 0.5 ps. The last 0.5 ps of each relaxation was used to estimate the free-energy profile, yielding 7.7 × 10^4^ structures in total, with their associated reaction coordinates and energy values. The QM energy values were distributed in groups of ~10^3^ different structures on average, along uniform 1D grid defined by the reaction coordinate. The partition function was calculated for each group in order to estimate a free-energy profile that was then smoothed via 2D LOESS local regression.

Error analysis was performed for 2D maps and 1D profiles using bootstrap resampling (100 replicates) on the data. In all relevant positions, the standard deviation was found below 0.8 kcal mol^−1^ for 2D maps and below 0.5 kcal mol^−1^ for 1D profiles. Standard deviation representation for 2D free-energy surface of ATP hydrolysis at AS1 in the presence of Rad21-Cter and for 1D free-energy profile of ATP hydrolysis at AS2 in the AS1-ADP/AS2-ATP condition can be found in the Supplementary Information (Supplementary Fig. 9).

### Free-energy difference calculations from trajectories of head domain separation

In order to compare the separation of Smc1A-head from Smc3-head, either ATP binding (AS1-ATP/AS2-ATP condition) or ADP binding (AS1-ADP/AS2-ADP condition) free-energy calculations from SMD trajectories were performed using Jarzynski’s equality^52^. Five individual SMD trajectories were generated for each condition, taking the ten initial structures from stable free MD trajectories. To prevent protein-protein collisions through the periodic boundaries, these were expanded and the water box was consequently enlarged with pre-equilibrated solvent cuboid boxes of TIP3P water model molecules. To ensure good thermalization of the system after the modification of the water box, a heating protocol similar to that used in the free MD simulations was applied. During each SMD trajectory, the centres of mass of Smc1A-head and Smc3-head were forced to separate 32.5 Å at a constant velocity over 13 ns (2.5 Å ns^−1^) with a spring constant of 5 kcal mol^−1^ Å^−2^, in the range of conditions used in similar SMD studies^72, 73^. The separation distance was kept constant for 0.1 ns at the start and end of each trajectory to better describe the quasi-equilibrium states at both ends. To avoid large rearrangements of the head structures during SMD, the Cα trace dihedrals were restrained with a 500 kcal mol^−1^ rad^−2^ force constant. The gap protection restraints used in the free MD simulations were also kept in the SMD simulations. For each calculation step, the distance between the centres of mass was recorded to later reconstruct the forces and work generated along each trajectory. The initial distance between the centres of mass was taken as the origin (0.0 Å) of separation in Fig. 4b and c.

## Electronic supplementary material

Supplementary Information

Supplementary Video 1

Supplementary Video 2

Supplementary Video 3

## References

[1] Haering, C. H. & Gruber, S. SnapShot: SMC Protein Complexes Part I. *Cell* **164**, 326–326 e321, doi:10.1016/j.cell.2015.12.026 (2016). 10.1016/j.cell.2015.12.026 26771499

[2] Uhlmann F (2016). SMC complexes: from DNA to chromosomes. Nat Rev Mol Cell Biol.

[3] Rankin, S. & Dawson, D. S. Recent advances in cohesin biology. *F1000Res* **5**, doi:10.12688/f1000research.8881.1 (2016). 10.12688/f1000research.8881.1 PMC4975370 27547382

[4] Gligoris T, Lowe J (2016). Structural Insights into Ring Formation of Cohesin and Related Smc Complexes. Trends Cell Biol.

[5] Haering CH (2004). Structure and stability of cohesin’s Smc1-kleisin interaction. Mol Cell.

[6] Huis in ‘t Veld PJ (2014). Characterization of a DNA exit gate in the human cohesin ring. Science.

[7] Gligoris TG (2014). Closing the cohesin ring: structure and function of its Smc3-kleisin interface. Science.

[8] Haering CH, Gruber S (2016). SnapShot: SMC Protein Complexes Part II. Cell.

[9] Liu J, Krantz ID (2009). Cornelia de Lange syndrome, cohesin, and beyond. Clin Genet.

[10] Mehta GD, Kumar R, Srivastava S, Ghosh SK (2013). Cohesin: functions beyond sister chromatid cohesion. FEBS Lett.

[11] Pie J (2016). Special cases in Cornelia de Lange syndrome: The Spanish experience. Am J Med Genet C Semin Med Genet.

[12] Gil-Rodriguez MC (2015). *De novo* heterozygous mutations in SMC3 cause a range of Cornelia de Lange syndrome-overlapping phenotypes. Hum Mutat.

[13] Watrin E, Kaiser FJ, Wendt KS (2016). Gene regulation and chromatin organization: relevance of cohesin mutations to human disease. Curr Opin Genet Dev.

[14] Ramos, F. J. *et al*. Clinical utility gene card for: Cornelia de Lange syndrome. *Eur J Hum Genet* **23**, doi:10.1038/ejhg.2014.270 (2015). 10.1038/ejhg.2014.270 PMC4592075 25537356

[15] Chetaille P (2014). Mutations in SGOL1 cause a novel cohesinopathy affecting heart and gut rhythm. Nat Genet.

[16] Izumi K (2015). Germline gain-of-function mutations in AFF4 cause a developmental syndrome functionally linking the super elongation complex and cohesin. Nat Genet.

[17] Mannini L, Menga S, Musio A (2010). The expanding universe of cohesin functions: a new genome stability caretaker involved in human disease and cancer. Hum Mutat.

[18] Pan XW (2016). SMC1A promotes growth and migration of prostate cancer *in vitro* and *in vivo*. Int J Oncol.

[19] Solomon DA, Kim JS, Waldman T (2014). Cohesin gene mutations in tumorigenesis: from discovery to clinical significance. BMB Rep.

[20] Krauthammer M (2012). Exome sequencing identifies recurrent somatic RAC1 mutations in melanoma. Nat Genet.

[21] Metzeler KH (2016). Spectrum and prognostic relevance of driver gene mutations in acute myeloid leukemia. Blood.

[22] Seshagiri S (2012). Recurrent R-spondin fusions in colon cancer. Nature.

[23] Hill VK, Kim JS, Waldman T (2016). Cohesin mutations in human cancer. Biochim Biophys Acta.

[24] Williams MS, Somervaille TC (2015). Leukemogenic Activity of Cohesin Rings True. Cell Stem Cell.

[25] De Koninck, M. & Losada, A. Cohesin Mutations in Cancer. *Cold Spring Harb Perspect Med* (in press); doi:10.1101/cshperspect.a026476 (2016). 10.1101/cshperspect.a026476 PMC5131750 27742736

[26] Bajic V, Spremo-Potparevic B, Zivkovic L, Isenovic ER, Arendt T (2015). Cohesion and the aneuploid phenotype in Alzheimer’s disease: A tale of genome instability. Neurosci Biobehav Rev.

[27] Ladurner R (2014). Cohesin’s ATPase activity couples cohesin loading onto DNA with Smc3 acetylation. Curr Biol.

[28] Murayama Y, Uhlmann F (2014). Biochemical reconstitution of topological DNA binding by the cohesin ring. Nature.

[29] Arumugam P (2003). ATP hydrolysis is required for cohesin’s association with chromosomes. Curr Biol.

[30] Weitzer S, Lehane C, Uhlmann F (2003). A model for ATP hydrolysis-dependent binding of cohesin to DNA. Curr Biol.

[31] Elbatsh AM (2016). Cohesin Releases DNA through Asymmetric ATPase-Driven Ring Opening. Mol Cell.

[32] Beckouet F (2016). Releasing Activity Disengages Cohesin’s Smc3/Scc1 Interface in a Process Blocked by Acetylation. Mol Cell.

[33] Murayama Y, Uhlmann F (2015). DNA Entry into and Exit out of the Cohesin Ring by an Interlocking Gate Mechanism. Cell.

[34] Lammens A, Schele A, Hopfner KP (2004). Structural biochemistry of ATP-driven dimerization and DNA-stimulated activation of SMC ATPases. Curr Biol.

[35] Arumugam P, Nishino T, Haering CH, Gruber S, Nasmyth K (2006). Cohesin’s ATPase activity is stimulated by the C-terminal Winged-Helix domain of its kleisin subunit. Curr Biol.

[36] Huber RG (2016). Impairing Cohesin Smc1/3 Head Engagement Compensates for the Lack of Eco1 Function. Structure.

[37] Kulemzina I (2016). A Reversible Association between Smc Coiled Coils Is Regulated by Lysine Acetylation and Is Required for Cohesin Association with the DNA. Mol Cell.

[38] Chao WC (2017). Structural Basis of Eco1-Mediated Cohesin Acetylation. Sci Rep.

[39] Li J, Feng W, Chen L, He J (2016). Downregulation of SMC1A inhibits growth and increases apoptosis and chemosensitivity of colorectal cancer cells. J Int Med Res.

[40] Wang J (2015). Role of SMC1A overexpression as a predictor of poor prognosis in late stage colorectal cancer. BMC Cancer.

[41] De Vivo M (2011). Bridging quantum mechanics and structure-based drug design. Front Biosci (Landmark Ed).

[42] Schramm VL (2013). Transition States, analogues, and drug development. ACS Chem Biol.

[43] Schramm VL (2015). Transition States and transition state analogue interactions with enzymes. Acc Chem Res.

[44] Mendieta-Moreno JI (2015). A Practical Quantum Mechanics Molecular Mechanics Method for the Dynamical Study of Reactions in Biomolecules. Adv Protein Chem Struct Biol.

[45] Mendieta-Moreno JI (2014). Fireball/amber: An Efficient Local-Orbital DFT QM/MM Method for Biomolecular Systems. J Chem Theory Comput.

[46] Case, D. A. *et al*. AMBER 14. University of California, San Francisco http://ambermd.org/ (2014).

[47] Lewis JP (2001). Further developments in the local-orbital density-functional-theory tight-binding method. Phys Rev. B.

[48] Lewis JP (2011). Advances and applications in the FIREBALL *ab initio* tight-binding moleculardynamics formalism. Phys. Status Solidi B.

[49] Marcos-Alcalde I, Setoain J, Mendieta-Moreno JI, Mendieta J, Gomez-Puertas P (2015). MEPSA: minimum energy pathway analysis for energy landscapes. Bioinformatics.

[50] Hayashi S (2012). Molecular mechanism of ATP hydrolysis in F1-ATPase revealed by molecular simulations and single-molecule observations. J Am Chem Soc.

[51] Yu H (2016). Magic Acts with the Cohesin Ring. Mol Cell.

[52] Jarzynski C (1997). Nonequilibrium equality for free energy differences. Phys Rev Lett.

[53] Wackerhage H (1998). Recovery of free ADP, Pi, and free energy of ATP hydrolysis in human skeletal muscle. J Appl Physiol (1985).

[54] Forbes SA (2015). COSMIC: exploring the world’s knowledge of somatic mutations in human cancer. Nucleic Acids Res.

[55] Gervasini C (2013). Cornelia de Lange individuals with new and recurrent SMC1A mutations enhance delineation of mutation repertoire and phenotypic spectrum. Am J Med Genet A.

[56] Ansari M (2014). Genetic heterogeneity in Cornelia de Lange syndrome (CdLS) and CdLS-like phenotypes with observed and predicted levels of mosaicism. J Med Genet.

[57] Mouradov D (2014). Colorectal cancer cell lines are representative models of the main molecular subtypes of primary cancer. Cancer Res.

[58] Kline AD (2007). Cornelia de Lange syndrome: clinical review, diagnostic and scoring systems, and anticipatory guidance. Am J Med Genet A.

[59] Mannini L, Liu J, Krantz ID, Musio A (2010). Spectrum and consequences of SMC1A mutations: the unexpected involvement of a core component of cohesin in human disease. Hum Mutat.

[60] Liu Y (2016). ATP-dependent DNA binding, unwinding, and resection by the Mre11/Rad50 complex. EMBO J.

[61] Schuler H, Sjogren C (2016). DNA binding to SMC ATPases-trapped for release. EMBO J.

[62] Seifert FU, Lammens K, Stoehr G, Kessler B, Hopfner KP (2016). Structural mechanism of ATP-dependent DNA binding and DNA end bridging by eukaryotic Rad50. EMBO J.

[63] Liu J (2009). SMC1A expression and mechanism of pathogenicity in probands with X-Linked Cornelia de Lange syndrome. Hum Mutat.

[64] Hopfner KP (2016). Invited review: Architectures and mechanisms of ATP binding cassette proteins. Biopolymers.

[65] Camdere G, Guacci V, Stricklin J, Koshland D (2015). The ATPases of cohesin interface with regulators to modulate cohesin-mediated DNA tethering. Elife.

[66] Kamada, K., Su’etsugu, M., Takada, H., Miyata, M. & Hirano, T. Overall Shapes of the SMC-ScpAB Complex Are Determined by Balance between Constraint and Relaxation of Its Structural Parts. *Structure*; doi:10.1016/j.str.2017.02.008 (2017). 10.1016/j.str.2017.02.008 28286005

[67] Hons MT (2016). Topology and structure of an engineered human cohesin complex bound to Pds5B. Nat Commun.

[68] Jorgensen W, Chandrasekhar J, Madura J, Impey R, Klein M (1983). Comparison of simple potential functions for simulating liquid water. J Chem Phys.

[69] Anandakrishnan R, Aguilar B, Onufriev AV (2012). H++ 3.0: automating pK prediction and the preparation of biomolecular structures for atomistic molecular modeling and simulations. Nucleic Acids Res.

[70] Mendieta-Moreno JI (2016). Quantum Mechanics / Molecular Mechanics Free Energy Maps and Nonadiabatic Simulations for a Photochemical Reaction in DNA: Cyclobutane Thymine Dimer. J Phys Chem Lett.

[71] Martin-Garcia F, Mendieta-Moreno JI, Lopez-Vinas E, Gomez-Puertas P, Mendieta J (2012). The Role of Gln61 in HRas GTP hydrolysis: a quantum mechanics/molecular mechanics study. Biophys J.

[72] Cheung LS (2015). Characterization of monobody scaffold interactions with ligand via force spectroscopy and steered molecular dynamics. Sci Rep.

[73] Kalyaanamoorthy S, Chen YP (2014). A steered molecular dynamics mediated hit discovery for histone deacetylases. Phys Chem Chem Phys.

[74] Kline AD (2007). Natural history of aging in Cornelia de Lange syndrome. Am J Med Genet C Semin Med Genet.

