# Supplementary material for: Two-step ATP-driven opening of cohesin head
Source: Sci Rep. 2017 Jun 12;7:3266. doi: 10.1038/s41598-017-03118-9 (PMC5468275; doi:10.1038/s41598-017-03118-9)
Supplement: Supplementary file 1 — Supplementary Information [file 41598_2017_3118_MOESM1_ESM.pdf]

Two-step ATP-driven opening of cohesin head

Íñigo Marcos-Alcalde, Jesús I. Mendieta-Moreno, Beatriz Puisac, María Concepción Gil-Rodríguez, María Hernández-Marcos, Diego Soler-Polo, Feliciano J. Ramos, José Ortega, Juan Pié, Jesús Mendieta, Paulino Gómez-Puertas

Supplementary Information

Containing Supplementary Figures 1-9 and Supplementary Videos 1-3.

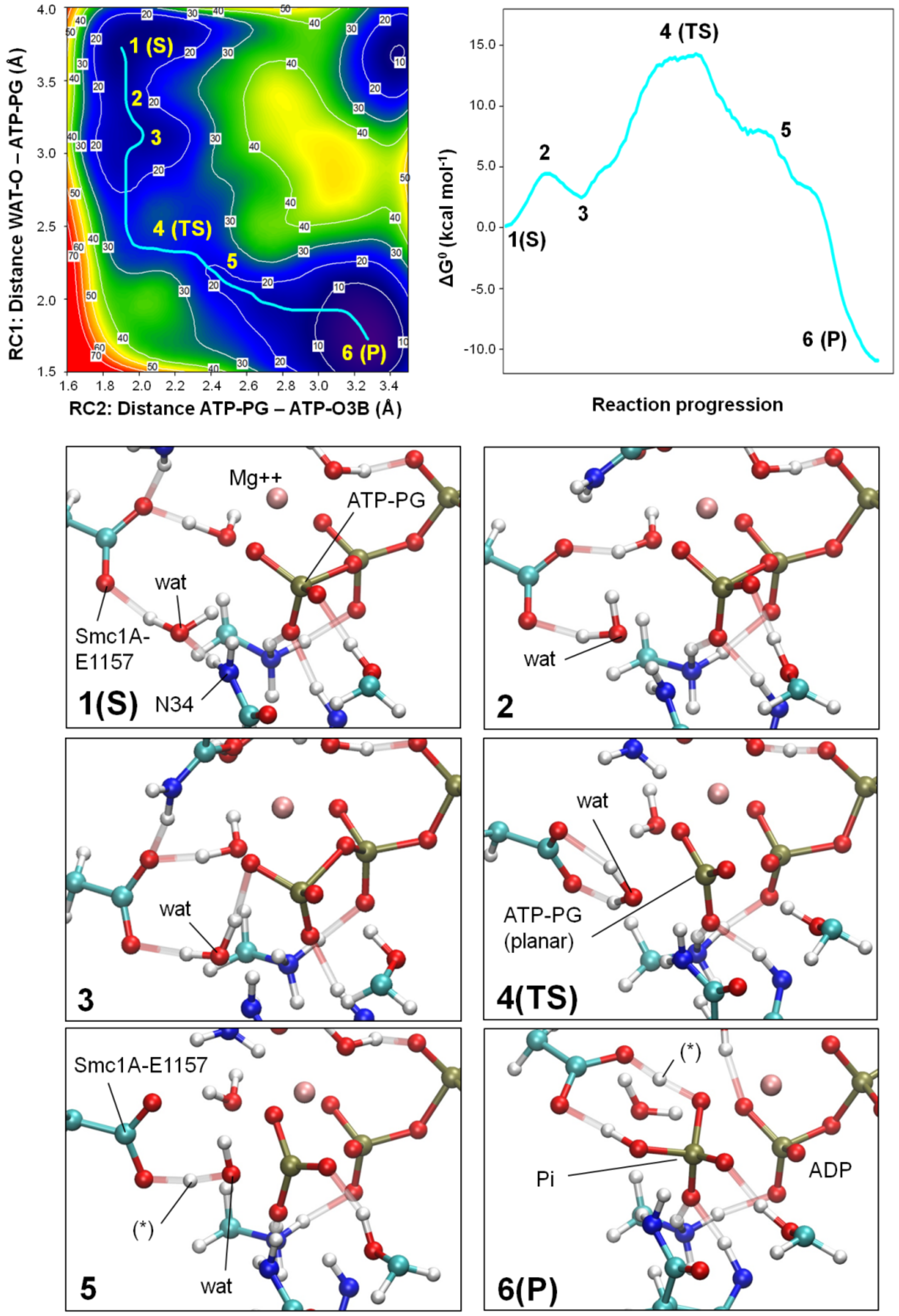

Supplementary Figure 1 | Points of interest in the energy profile of the ATP hydrolysis at AS1 in the presence of Rad21-Cter. Points of interest (1-6) of the MEPSA minimum energy path are indicated on the energy surface and the energy profile representation. A reference structure is shown for each. Frames 1 to 3 illustrate the initial water stabilization via hydrogen bonding with Smc1A-N34 (1), which is subsequently swapped for hydrogen bonding with the γ-phosphate (3), through an intermediate transition state (2). After the highest free-energy barrier associated with the planar configuration in the main transition state (4) is overcome, a small free-energy barrier associated with catalytic water deprotonation by Smc1A-E1157 can be observed (5). The product structure (6) is reached when the leaving inorganic phosphate group (Pi) is formed and when Smc1A-E1157 transfers the catalytic water proton to the leaving group. The proton (*) initially transferred from the catalytic water to Smc1A-E1157 in (5) is labelled to illustrate the rotation of the Smc1A-E1157 residue prior to transfer of the proton to the inorganic phosphate (6).

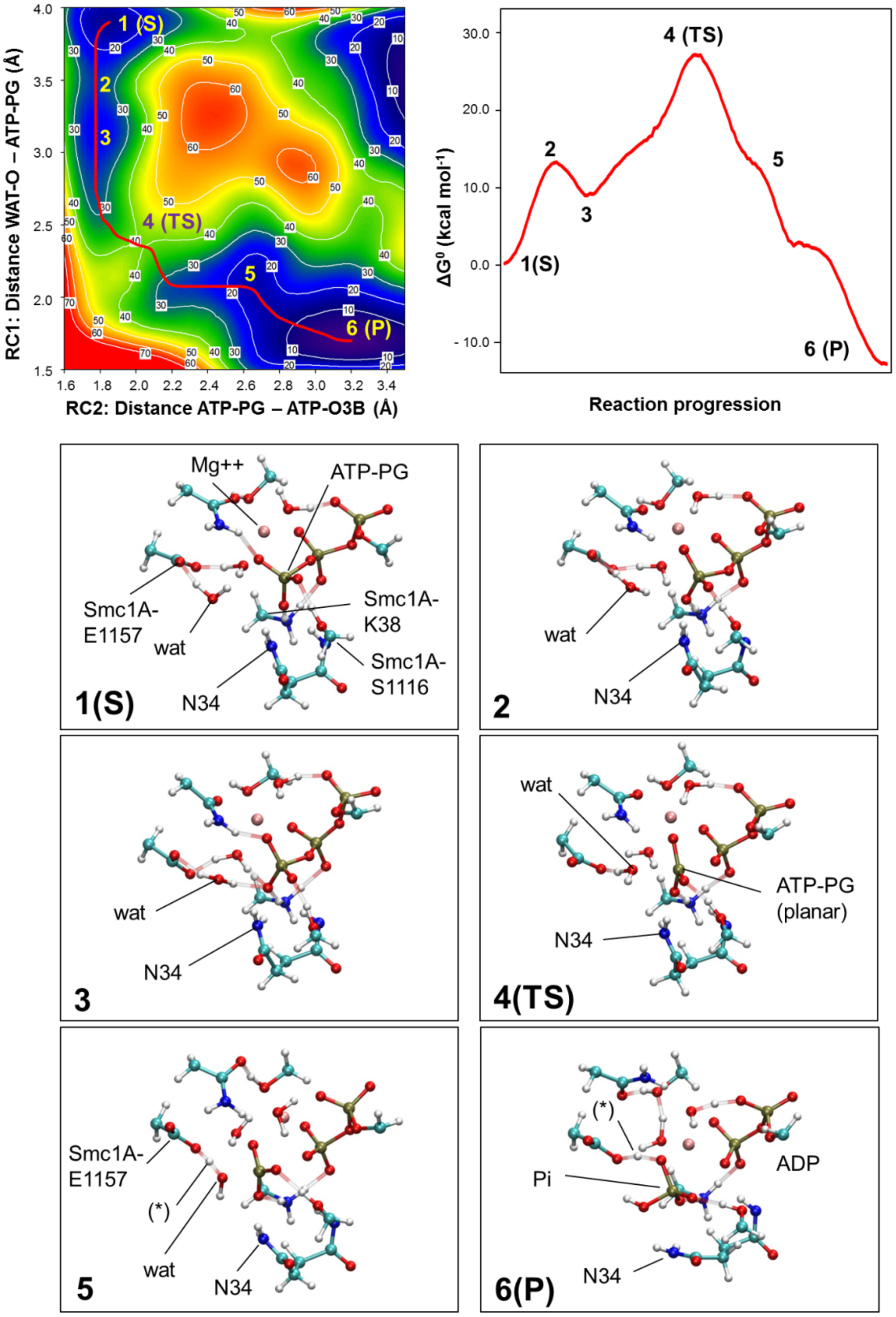

Supplementary Figure 2 | Points of interest in the energy profile of the ATP hydrolysis at AS1 in the absence of Rad21-Cter. Points of interest (1-6) of the MEPSA minimum energy path are indicated on the energy surface and the energy profile representation. A reference structure is shown for each. Frames 1 to 3 illustrate the initial water placement, from the initial location (1) to the stabilization via hydrogen bonding with the γ-phosphate (3), through the intermediate transition state (2). Note the location of Smc1A-N34, far from the water position. Structures from the main transition state (4), catalytic water deprotonation event (5) and product (6) are also displayed. The proton (*) transferred from the catalytic water to Smc1A-E1157 in (5) is labelled.

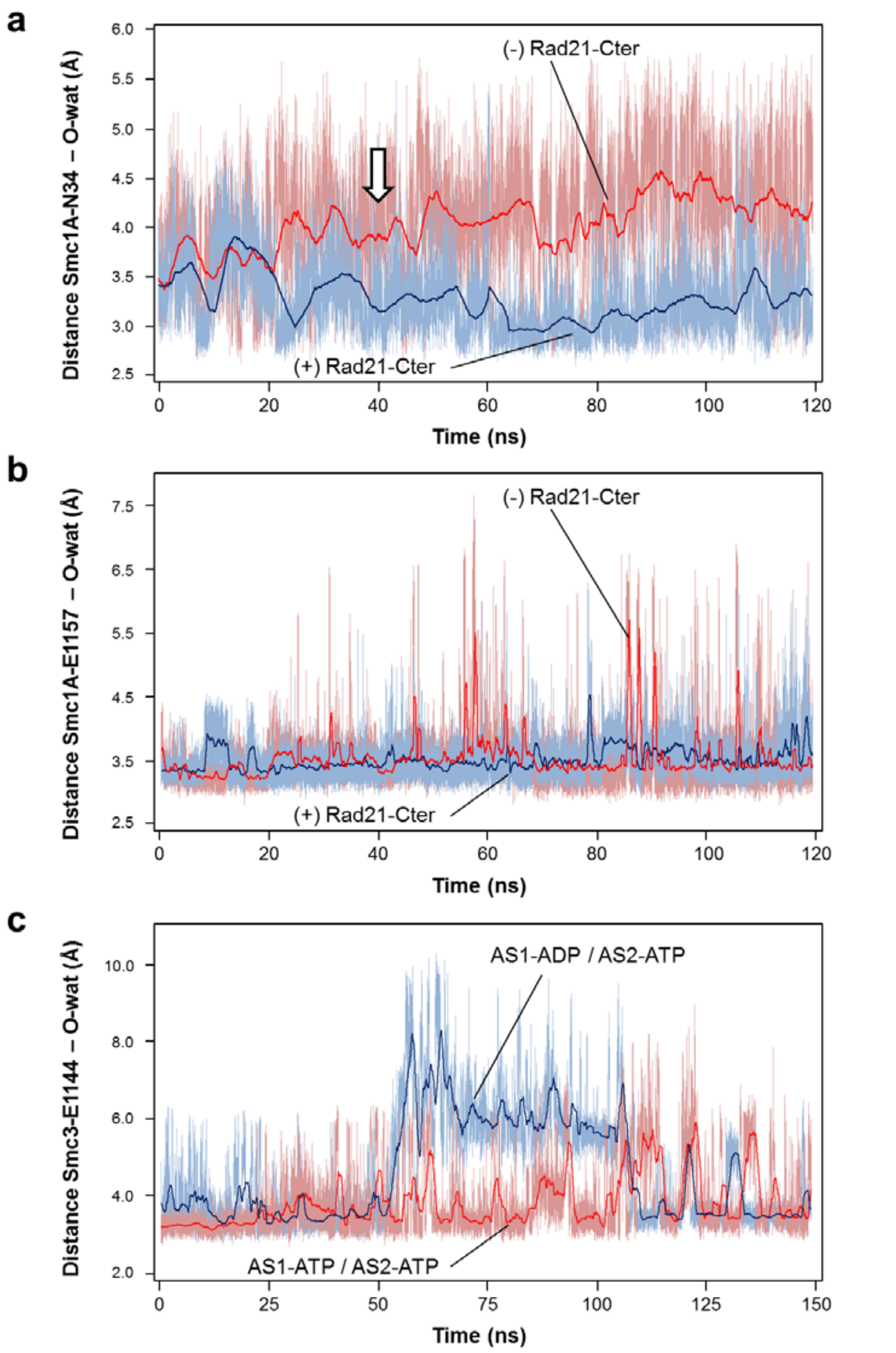

Supplementary Figure 3 | Position of catalytic water molecules in the active centres. (a) Evolution of the distance between the oxygen atom of the catalytic water in AS1 and the Cγ atom of the Smc1A-N34 residue (distance Smc1A-N34 - wat-O) in presence (cyan) and absence (red) of Rad21-Cter. The arrow indicates the position of the two snapshots selected as initial structure for QM/MM MD simulations. **(b)** Evolution of the distance between the oxygen atom of the catalytic water in AS1 and the Cδ atom of the catalytic Smc1A-E1157 residue (distance Smc1A-E1157 - wat-O) in presence (cyan) and absence (red) of Rad21-Cter. **(c)** Evolution of the distance between the oxygen atom of the catalytic water in AS2 and the Cδ atom of the catalytic Smc3-E1144 residue (distance Smc3-E114 - wat-O) prior (red) and after (cyan) ATP hydrolysis at AS1. The movement of the Smc3-E1144 residue, coordinated to the reallocation of Smc1A-K1120 in AS2, can be visualized in Supplementary video 2.

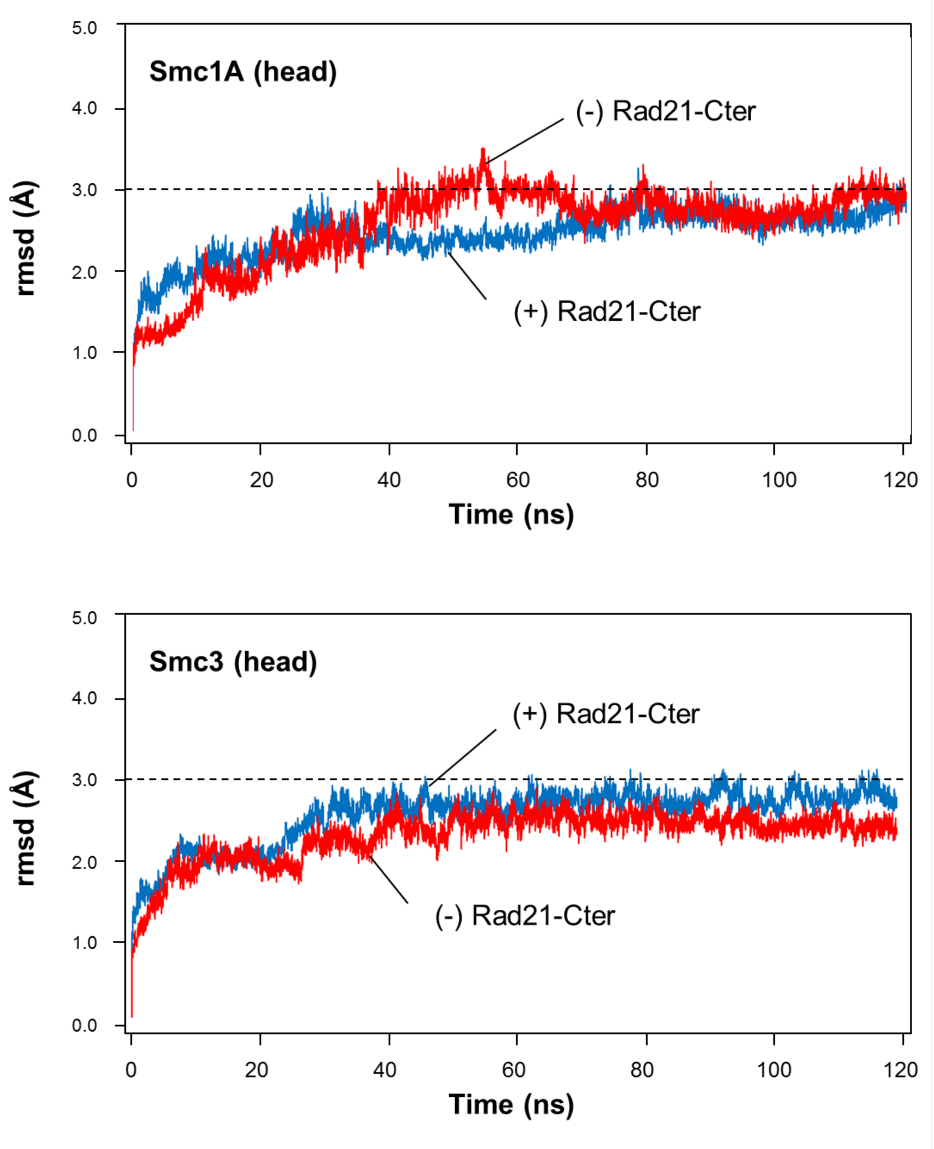

Supplementary Figure 4 | Root mean square deviation (rmsd) values of Smc1A-head and Smc3-head domains. rmsd values measured over the unrestricted 120 ns MD trajectory of the cohesin head complex in presence (cyan) and absence (red) of the Rad21-Cter domain.

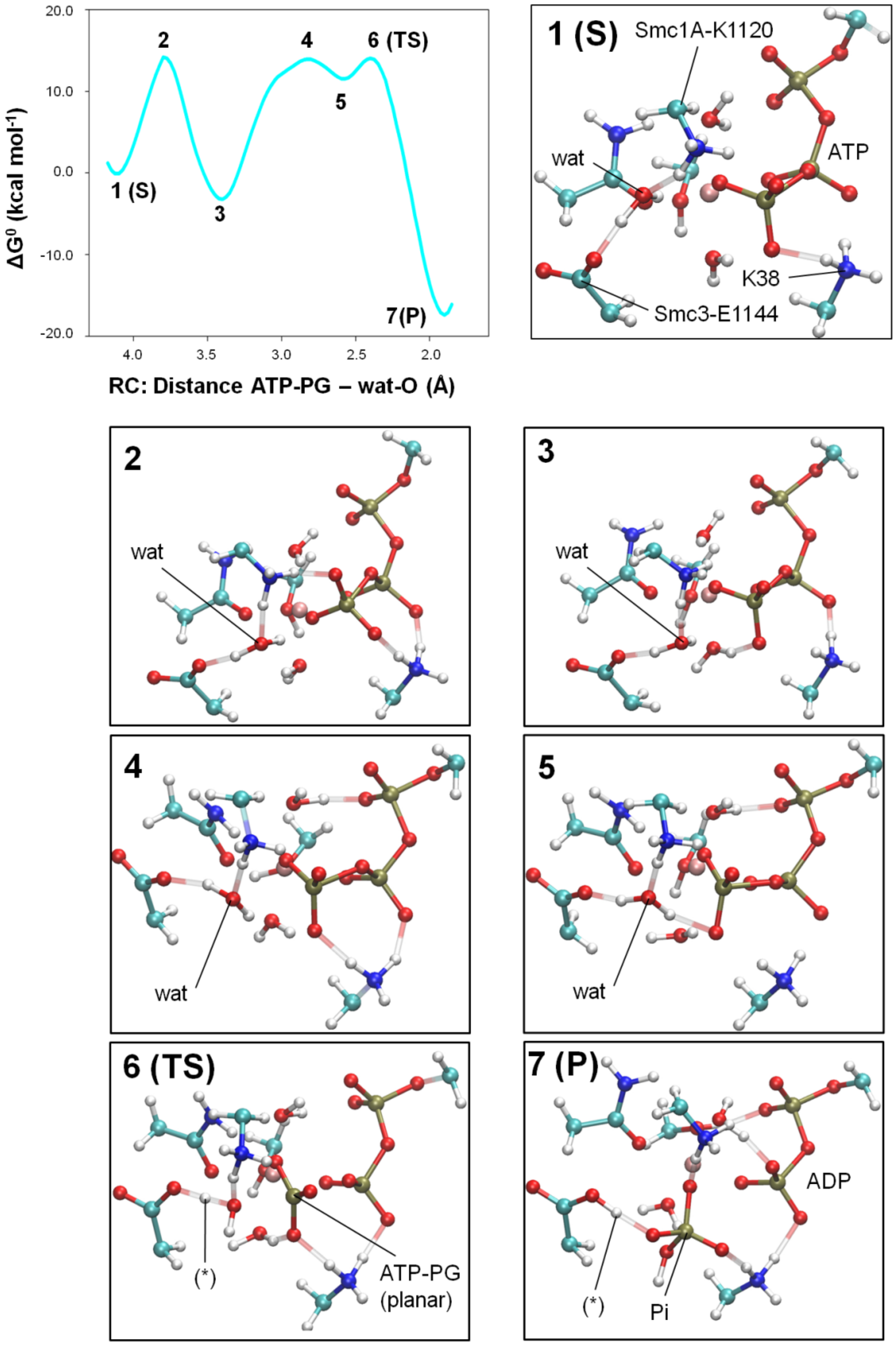

Supplementary Figure 5 | Points of interest in the energy profile of the ATP hydrolysis at AS2 in its active form (AS1-ADP/AS2-ATP). Points of interest (1-7) of the free-energy profile are indicated and a reference structure is shown for each one. The energy profile can be divided into 3 steps: the first (panels 1 to 3), is characterized by the entrance of the catalytic water molecule in the active site; the second (panels 3 to 5), corresponds to the stabilization of the catalytic water molecule prior to the transition state by triple hydrogen bonding with Smc3-E1144, Smc1A-K1120 and γ-phosphate; the final one (panels 6 and 7) correlates to the transition state, thus leading to the product structure. The leaving inorganic phosphate group is formed and Smc3-E1144 transfers the catalytic water proton (*) to the leaving group.

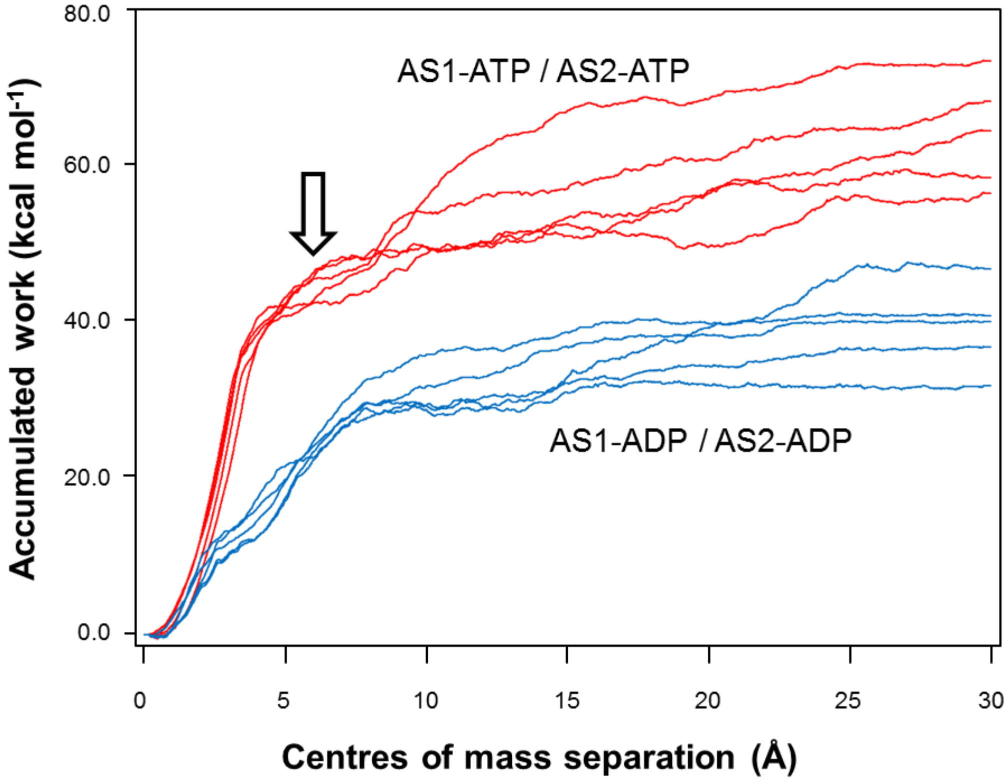

Supplementary Figure 6 | Accumulated work (kcal mol^{-1}) along the independent trajectories over the separation between the centres of mass of the Smc1A-head and Smc3-head domains. Points from all five SMD trajectories for the AS1-ATP/AS2-ATP condition (red) and for AS1-ADP/AS2-ADP condition (cyan) are shown. The arrow indicates the distance in which the residues in both sides of the two active centres are completely detached.

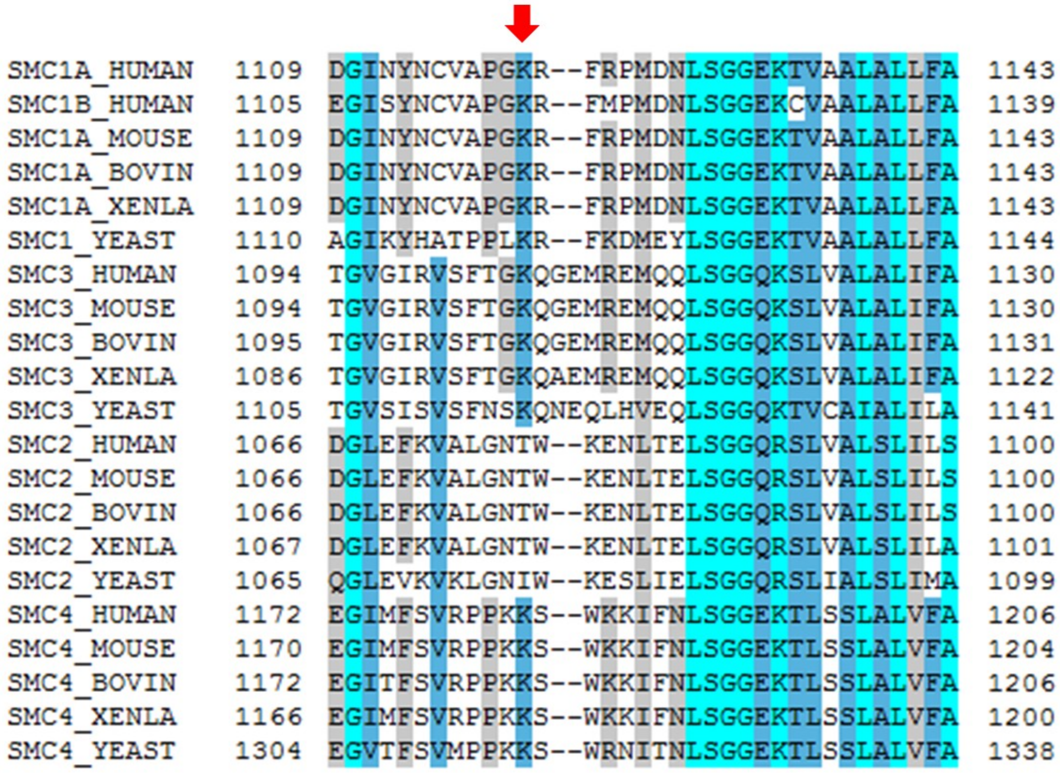

Supplementary Figure 7 | Multiple sequence alignment of several proteins homologous to human Smc1A in the area surrounding residue K1120. The sequences represented are: human Smc1A (SMC1A_HUMAN), Smc1B (SMC1B_HUMAN), Smc3 (SMC3_HUMAN), Smc2 (SMC2_HUMAN) and Smc4 (SMC4_HUMAN); *Mus musculus* Smc1A (SMC1A_MOUSE), Smc3 (SMC3_MOUSE), Smc2 (SMC2_MOUSE) and Smc4 (SMC4_MOUSE); *Bos taurus* Smc1A (SMC1A_BOVIN), Smc3 (SMC3_BOVIN), Smc2 (SMC2_BOVIN) and Smc4 (SMC4_BOVIN); *Xenopus laevis* Smc1A (SMC1A_XENLA), Smc3 (SMC3_XENLA), Smc2 (SMC2_XENLA) and Smc4 (SMC4_XENLA); and *Saccharomyces cerevisiae* Smc1 (SMC1_YEAST), Smc3 (SMC3_YEAST), Smc2 (SMC2_YEAST) and Smc4 (SMC4_YEAST). The residues are coloured according to conservation (BLOSUM62 score). The position of human Smc1A-K1120 is indicated by an arrow.

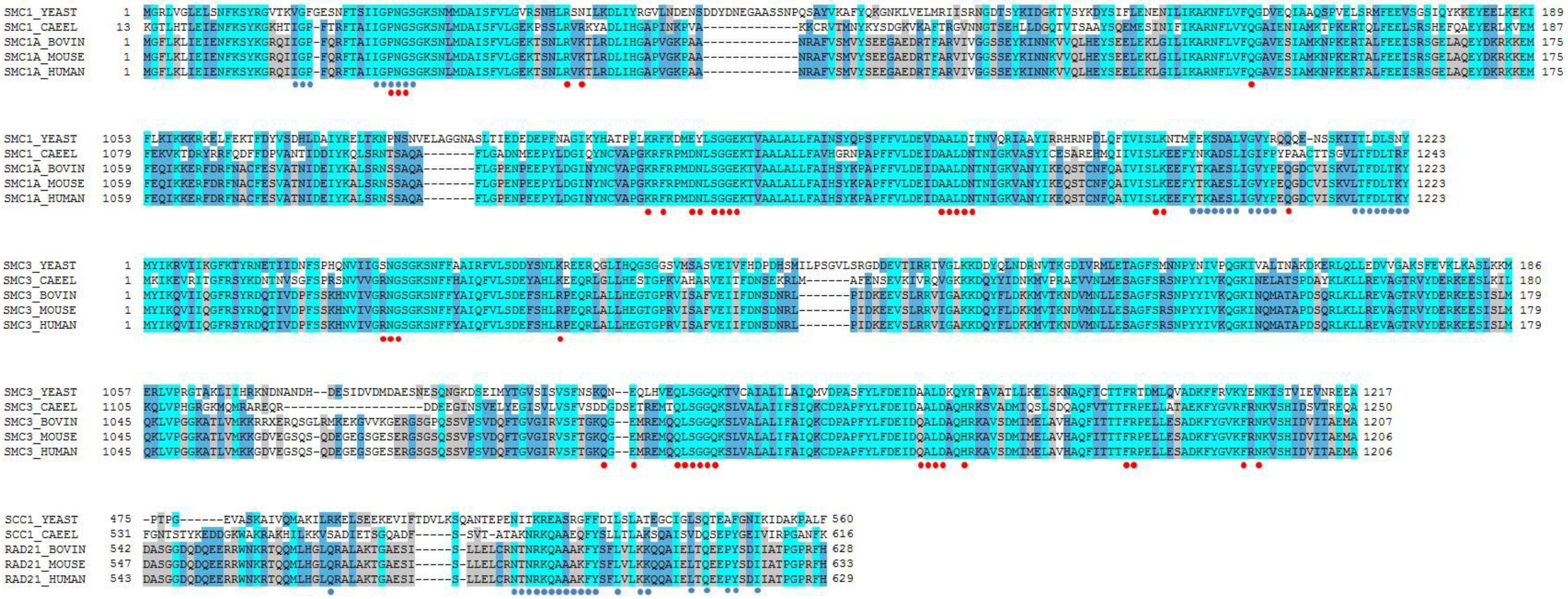

Supplementary Figure 8 | Multiple sequence alignment of the modelled sequences. The sequences represented are: human Smc1A (SMC1A_HUMAN), Smc3 (SMC3_HUMAN) and Rad21 (RAD21_HUMAN); *Mus musculus* Smc1A (SMC1A_MOUSE), Smc3 (SMC3_MOUSE) and Rad21 (RAD21_MOUSE); *Bos taurus* Smc1A (SMC1A_BOVIN), Smc3 (SMC3_BOVIN) and Rad21 (RAD21_BOVIN); *Caenorhabditis elegans* Smc1 (SMC1_CAEEL), Smc3 (SMC3_CAEEL) and Scc1 (SCC1_CAEEL); and *Saccharomyces cerevisiae* Smc1 (SMC1_YEAST), Smc3 (SMC3_YEAST) and Scc1 (SCC1_YEAST). The residues are coloured according to conservation (BLOSUM62 score). Contacting residues in the interfaces between Smc1A/Smc3 (red dots) and Smc1A/Rad21-Cter (blue dots) are highlighted, locating all of them preferentially in the most conserved regions of the alignment.

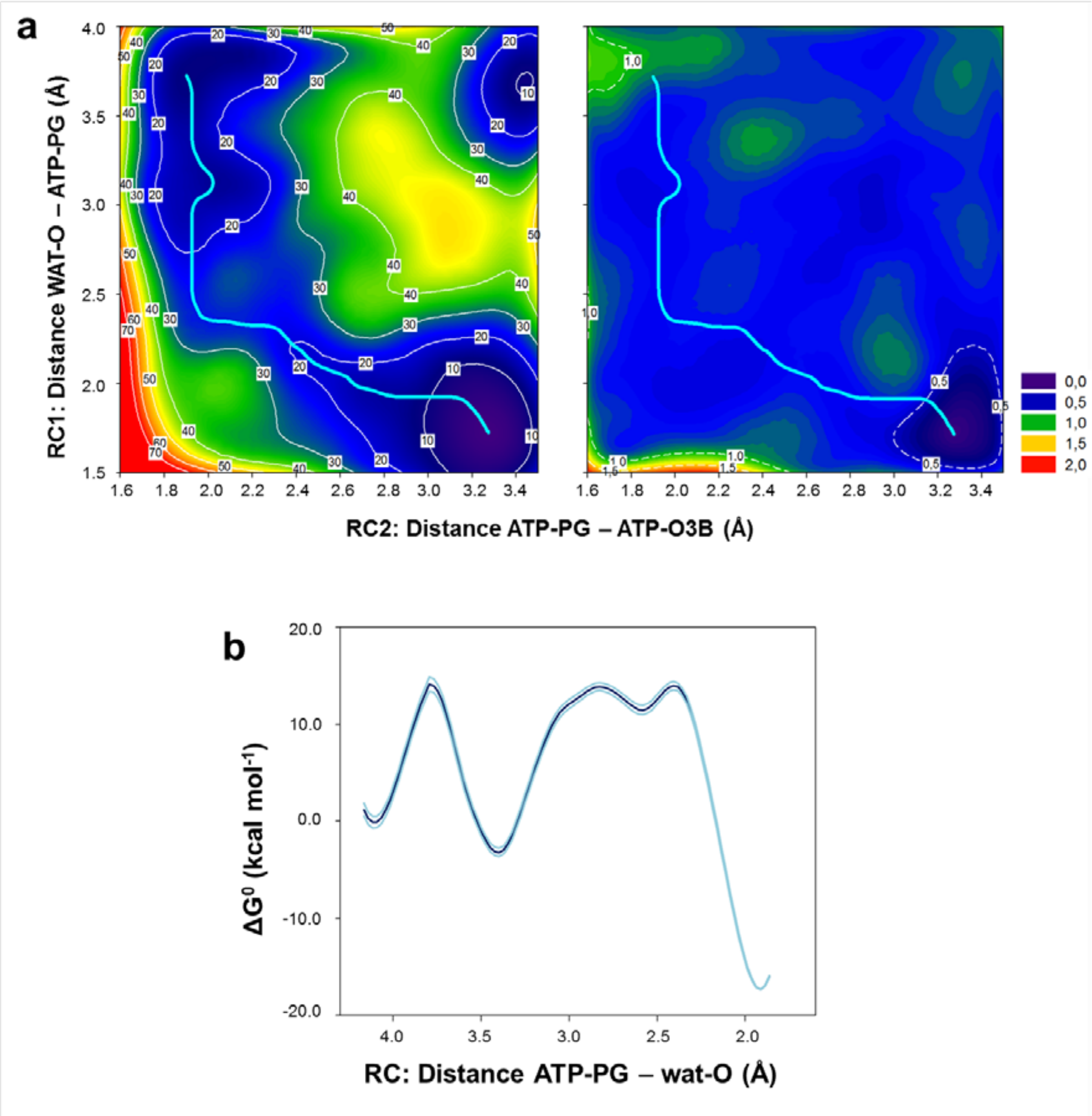

Supplementary Figure 9 | Error analysis of free energy values. (a) Standard deviation values (right) of the free-energy surface (left) for ATP hydrolysis at AS1 in the presence of Rad21-Cter. Error analysis was performed using bootstrap resampling (100 replicates) on the data. Color scale for standard deviation values is included. **(b)** Mean values +/- standard deviation of the 1D free-energy profile of ATP hydrolysis at AS2 in the AS1-ADP/AS2-ATP condition. Error analysis was performed using bootstrap resampling (100 replicates) on the data.

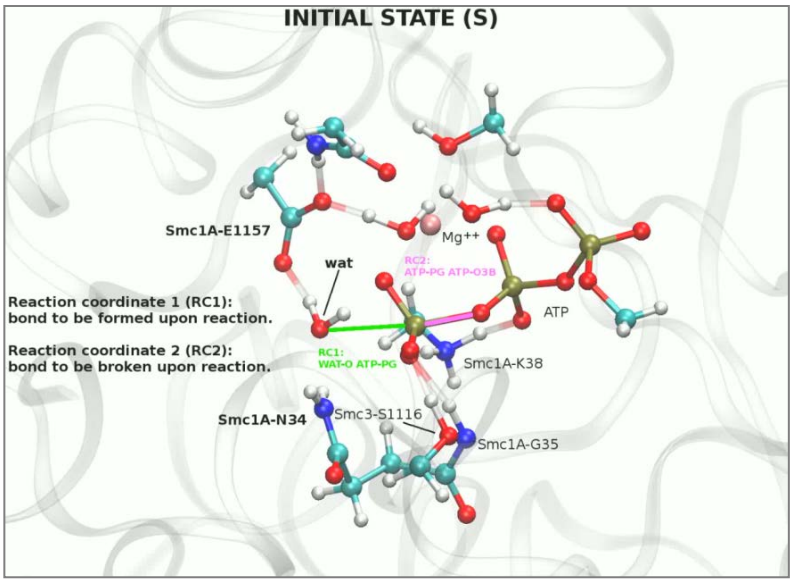

Supplementary Video 1 | ATP hydrolysis at AS1 in the presence of Rad21. The reaction along the MEPSA minimum energy path is shown in Supplementary Fig. 1. The different steps in the reaction are highlighted: initial structure (S), stabilization of the catalytic water molecule, transition state (TS) and final product (P).

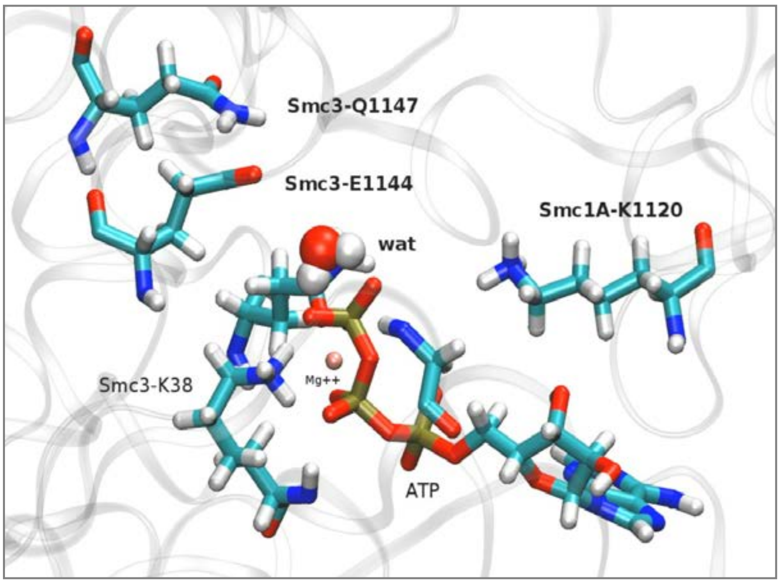

Supplementary Video 2 | Positioning of Smc1A-K1120. After ATP hydrolysis at AS1, Smc1A-K1120 moves close to the AS2 catalytic water molecule and remained in its new location in a stable conformation. The movie shows the MD from time 75 to 150 ns of Fig. 3a. Position of residue Smc3-Q1147 is also indicated. Protons are not shown during movement to avoid smoothing artifacts.

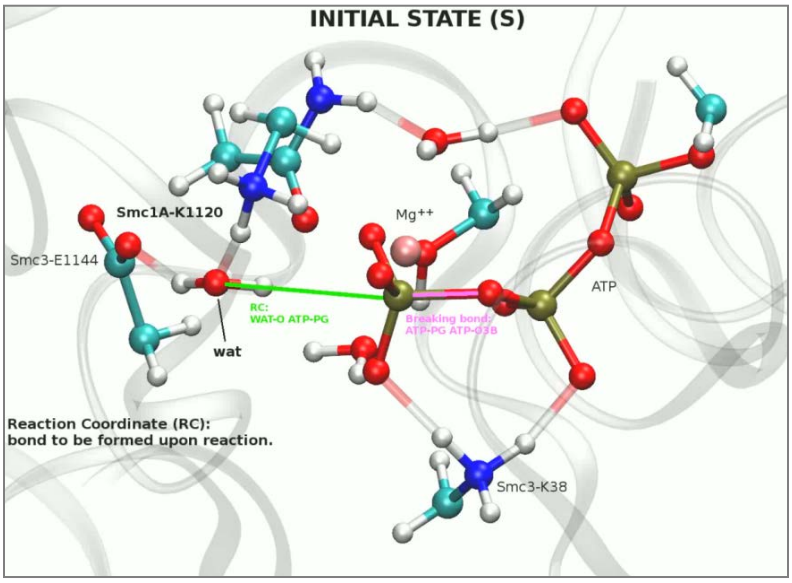

Supplementary Video 3 | ATP hydrolysis at AS2 in its active form (AS1-ADP/AS2-ATP). The reaction along the RC1 coordinate as indicated in Supplementary Fig. 5. Sequential steps in the reaction are highlighted: initial structure (S), transition state (TS) and final product (P).
